# TOLLIP Inhibits Psoriasis Progression via Suppressing PKM2‐Mediated Glycolysis in Keratinocytes

**DOI:** 10.1002/advs.202523390

**Published:** 2026-07-31

**Authors:** Xiuhuan Jiang, Guoying Miao, Na Shen, Qingxia Han, Xiaoru Han, Zichang Qiao, Kexin Feng, Zhuo Tan, Yaguang Wang, Xiuhua Liu, Chen Wu, Zhenzhen Yan

**Affiliations:** ^1^ Baoding Key Laboratory of Cancer & Aging College of Life Science Hebei University Baoding China; ^2^ Department of Dermatology Affiliated Hospital of Hebei Engineering University Handan China; ^3^ State Key Laboratory of New Pharmaceutical Preparations and Excipients Hebei University Baoding China

**Keywords:** CUE, Glycolysis, PKM2, Psoriasis, TOLLIP

## Abstract

Metabolic reprogramming toward aerobic glycolysis is increasingly recognized as a key mechanism in the pathogenesis of psoriasis, but the underlying regulatory mechanisms remain unclear. Here, we identify Toll‐interacting protein (TOLLIP) as a critical regulator of psoriasis pathogenesis through its modulation of glycolytic metabolism. We found that TOLLIP is significantly upregulated in psoriatic lesions, and its genetic deletion in mice exacerbated disease severity in imiquimod (IMQ)‐induced psoriasis models. Mechanistically, TOLLIP interacts with the rate‐limiting glycolytic enzyme pyruvate kinase M2 (PKM2) via its coupling of ubiquitin to ER degradation (CUE) domain (179–274 aa), inhibits PKM2's metabolic enzyme activity and attenuates aerobic glycolysis, thereby mitigating keratinocyte hyperproliferation and inflammation. Therapeutic delivery of Tollip or Tollip (179–274 aa) via adeno‐associated virus (AAV) vectors ameliorated psoriasis progression in mice. Our findings establish the TOLLIP‐PKM2‐glycolysis axis as a key mechanism linking metabolic reprogramming to psoriasis pathogenesis, and propose TOLLIP as a promising therapeutic target.

## Graphical abstract

In this study, we identify TOLLIP as a critical regulator of psoriasis pathogenesis through its modulation of glycolytic metabolism. Our findings establish the TOLLIP‐PKM2‐glycolysis axis as a key mechanism linking metabolic reprogramming to psoriasis pathogenesis, and propose TOLLIP as a promising therapeutic target.

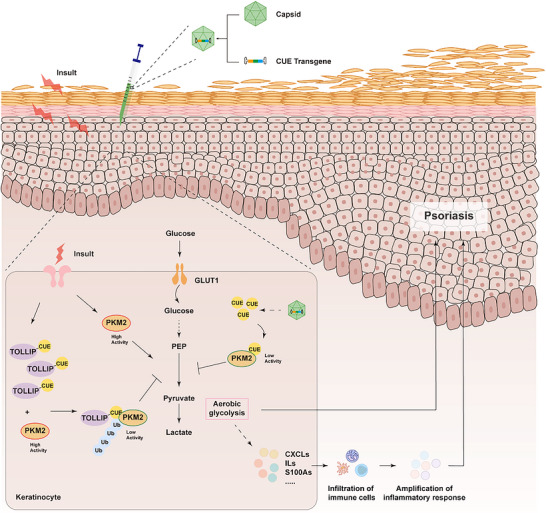

## 1 Introduction

Psoriasis is a chronic inflammatory dermatosis affecting approximately 2%–3% of the global population [1]. Characterized by erythematous, scaly plaques accompanied by pruritus and epidermal thickening, this condition profoundly impairs patients' quality of life and psychosocial health [2]. Beyond its cutaneous manifestations, psoriasis is increasingly recognized as a systemic disorder, with growing evidence linking it to multiple comorbidities, including cardiovascular diseases, metabolic syndrome, non‐alcoholic fatty liver disease, respiratory dysfunction, psychiatric conditions, and concurrent autoimmune inflammatory disorders [3, 4, 5, 6, 7]. This multifaceted disease burden underscores psoriasis as a significant public health challenge worldwide.

At the molecular level, psoriasis is characterized by excessive keratinocyte proliferation, aberrant differentiation, and inflammatory cell infiltration [8, 9]. Keratinocytes, the predominant cellular component of the epidermis, function as critical signal transducers, converting environmental stimuli into robust production of proinflammatory cytokines, antimicrobial peptides (AMPs), and chemotactic factors, which initiate cutaneous inflammation by recruiting and activating immune cell populations such as T cells and dendritic cells [10]. In turn, these immune cells release cytokines including IL‐17, IL‐22, and TNF‐α, which further stimulate keratinocytes and exacerbate the inflammatory response. This reciprocal crosstalk generates a self‐amplifying inflammatory loop that drives both pathological epidermal hyperplasia and a reinforced inflammatory microenvironment [2, 7].

Emerging evidence indicates that psoriatic keratinocytes undergo significant metabolic reprogramming, particularly through enhanced aerobic glycolysis (the Warburg effect), elevated glucose uptake, and increased lactate production. This metabolic shift is not merely an energy‐supplying adaptation but an active regulatory process that supports rapid biomass synthesis during proliferation and fuels inflammatory signaling [11, 12, 13]. Recent studies have reported a broad upregulation of glycolysis‐related proteins in psoriatic keratinocytes, which is positively associated with disease severity [10, 11, 14]. For example, GLUT1, the predominant glucose transporter in epidermal keratinocytes, facilitates glucose influx to drive the anabolic demands of hyperproliferation [15]; PFKFB3, the most potent allosteric activator of the rate‐limiting enzyme PFK‐1, sustains the hyperproliferative and poorly differentiated phenotype of psoriatic epidermis [14]; and PKM2 drives aerobic glycolysis and keratinocyte proliferation while also directly regulating NF‐κB signaling downstream of the IL‐17 receptor through its non‑metabolic enzyme activity, thereby initiating cutaneous psoriatic inflammation [11, 16]. Furthermore, pro‑inflammatory cytokines such as IFNγ and TNF‑α have been shown to directly induce glycolytic reprogramming in keratinocytes, linking immune signals to metabolic adaptation [17]. Together, these findings illustrate how intrinsic metabolic reprogramming in keratinocytes interfaces with and amplifies the cytokine signaling network, with both processes converging to sustain the psoriatic phenotype. Despite these advances, the upstream regulators that orchestrate the activity of these key glycolytic enzymes, and whether novel molecules exist to modulate this pathogenic metabolic hub, remain to be fully elucidated.

Toll‐interacting protein (TOLLIP), first identified in 2000 as an interacting partner of interleukin‐1 receptor accessory protein [18], is a 274‐amino acid adaptor protein characterized by three functional domains: an N‐terminal Tom1‐binding domain (TBD), a central conserved C2 domain, and a C‐terminal coupling of ubiquitin to ER degradation (CUE) domain [19]. As a ubiquitously expressed regulatory protein, TOLLIP plays a critical role in modulating various cellular processes, including inflammatory signaling, autophagic flux, and vesicular trafficking [19, 20, 21]. Emerging evidence indicates that TOLLIP also plays a role in cellular metabolism. For instance, TOLLIP has been shown to inhibit lipid accumulation in macrophages, and its deficiency is associated with systemic metabolic alterations, including increased circulating lipids and liver steatosis [22, 23]. Notably, TOLLIP promotes the degradation of monocarboxylate transporter 1 (MCT1), a key transporter of lactate—the end‐product of aerobic glycolysis—suggesting that TOLLIP may serve as an upstream regulator of glycolytic flux [24]. Given that psoriasis progression is associated with aerobic glycolysis, we hypothesize that TOLLIP may play a role in psoriasis by modulating the metabolic reprogramming of keratinocytes. However, whether and how TOLLIP regulates glycolytic reprogramming in psoriatic keratinocytes remains unknown. This study aims to investigate whether and how TOLLIP regulates glycolytic reprogramming in keratinocytes and to elucidate its impact on psoriasis pathogenesis.

Here, we identify TOLLIP as a novel regulator of psoriasis that acts by constraining PKM2‐mediated aerobic glycolysis. Integrated analysis of public transcriptomic datasets and experimental validation revealed a marked upregulation of TOLLIP in psoriatic lesions. Functional studies demonstrated that *Tollip* knockout (KO) mice showed aggravated epidermal hyperplasia, inflammation, and disease severity in an IMQ‐induced psoriasis‐like skin model. Keratinocyte‐specific re‐expression of Tollip could ameliorate the exacerbated psoriatic phenotype in *Tollip*‐KO mice. Overexpression of TOLLIP in HaCaT keratinocytes reduced cytokine‐induced cell proliferation and inflammation, whereas knockdown of TOLLIP yielded opposite results. Consistently, primary keratinocytes isolated from wild‐type and *Tollip*‐KO mice showed similar results in regulating keratinocytes’ proliferation and inflammation. Guided by re‐analysis of published interactome data, we mechanistically delineate that TOLLIP binds to the rate‐limiting glycolytic enzyme PKM2 via its CUE (179‐274 aa) domain. This interaction promotes PKM2 ubiquitination, which inhibits its enzymatic activity and, in turn, suppresses glycolytic flux. Importantly, therapeutic targeting of this axis via AAV‐mediated delivery of Tollip or Tollip (179–274 aa) significantly ameliorates psoriasis manifestations in mice. Our findings establish TOLLIP as a novel negative regulator of psoriasis that acts by constraining PKM2‐mediated glycolysis, highlighting the therapeutic potential of targeting the TOLLIP–PKM2–glycolysis axis.

## 2 Results

### 2.1 The Expression of TOLLIP Is Associated With Psoriasis Severity

**FIGURE 1:**
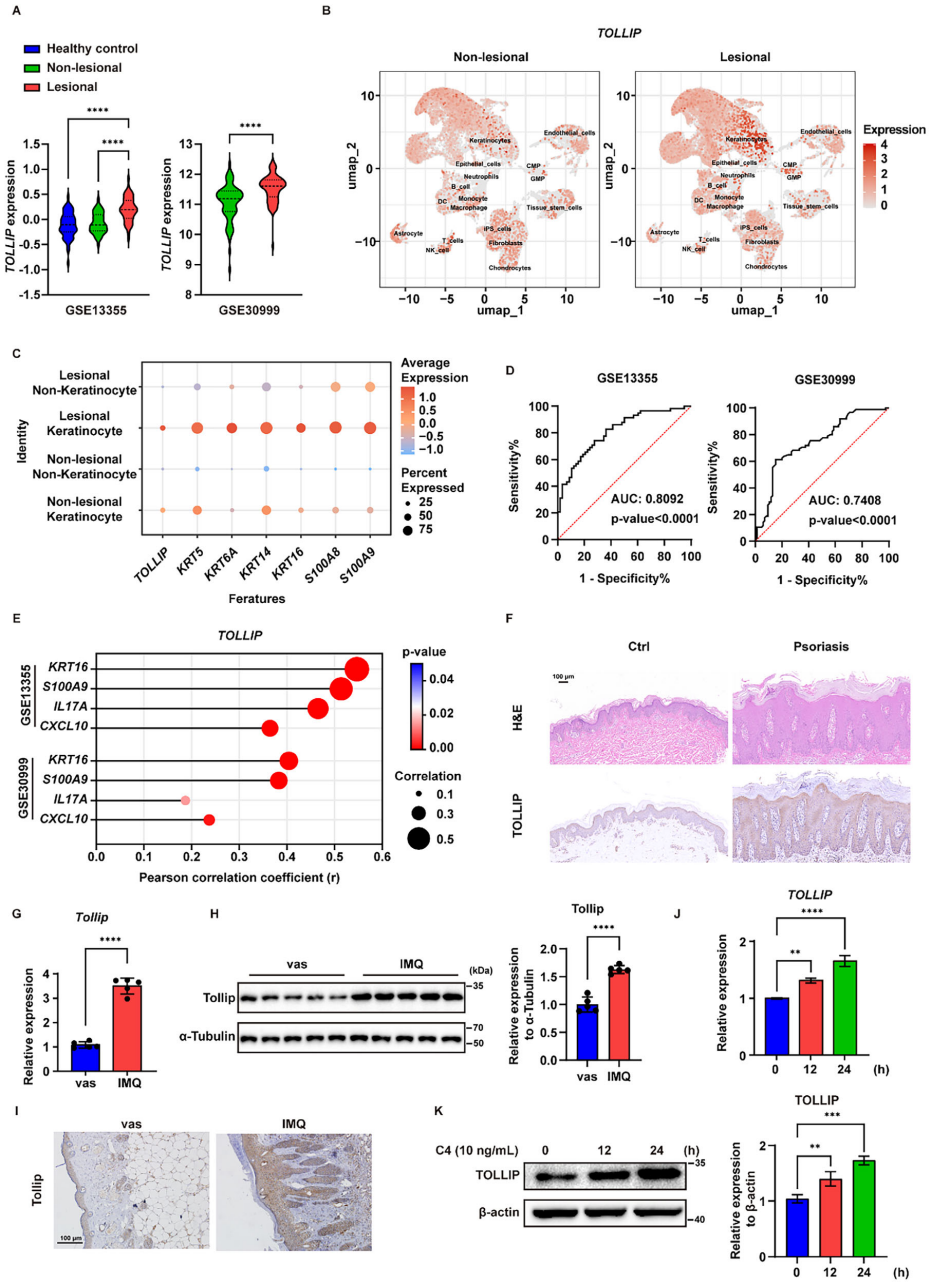
Correlation between TOLLIP expression and psoriasis severity. (A) Expression levels of *TOLLIP* in skin biopsy samples from healthy controls, non‐lesional skin and lesional skin of psoriatic patients, as assessed through analyzing the transcriptome of published datasets: GSE13355 (healthy control, *n* = 64; non‐lesional, *n* = 58; lesional, *n* = 58), GSE30999 (healthy control, *n* = 85; lesional, *n* = 85). (B) Feature plot (GSE228421) showing expression profiles of *TOLLIP* (red) in skin cell groups. The red gradient indicates the normalized expression level of the *TOLLIP* gene, ranging from 0 to 4. (C) Dot plot of *TOLLIP* and keratinocyte marker gene expression from single‐cell RNA sequencing data (GSE228421) in the indicated four cell subgroups. The size of each dot denotes the percentage of cells expressing the corresponding gene within each group, while the color gradient reflects the average gene expression level calculated from all gene‐positive cells; a deeper reddish color indicates higher average transcriptional abundance of the target gene. (D) Diagnostic performance of *TOLLIP* for psoriasis in GSE13355 and GSE30999. (E) Pearson correlation analysis was performed between the expression of *TOLLIP* and *KRT16*, *S100A9*, *IL17A*, and *CXCL10* genes in skin transcriptomic datasets: GSE13355, GSE30999. (F) Representative immunohistochemical staining image of TOLLIP expression in the epidermis of control subjects (Ctrl, *n* = 5) and psoriasis patients (*n* = 5). Scale bars, 100 µm. (G) qPCR analysis of *Tollip* mRNA expression in skin samples from mice treated with control cream or IMQ for 5 days. *Tuba1a* was used as a reference gene for normalization (*n* = 5 mice per group). (H) Western blot analysis (left) and quantification (right) of Tollip protein levels in skin tissues from mice treated with control cream (vaseline, vas) or imiquimod (IMQ) for 5 days. α‐Tubulin was used as a loading control (*n* = 5 mice per group). (I) Representative immunohistochemistry images of dorsal skin sections stained for Tollip following 5‐day treatment with control cream (vas) or IMQ (*n* = 3 mice per group). Scale bar, 100 µm. (J) qPCR analysis of *TOLLIP* mRNA expression in HaCaT cells after C4, a cytokine cocktail (IL‐17A, IL‐22, TNF‐α, and OSM, 10 ng/mL) application for the indicated times. *ACTB* was used as a reference gene for normalization. (K) Western blot analysis (left) and quantification (right) of TOLLIP protein expression in HaCaT cells after C4 stimulation for the indicated times. β‐actin was used as a loading control. Data represent three independent experiments and are shown as mean ± SD. ^**^ *p* < 0.01, ^***^ *p* < 0.001, ^****^ *p* < 0.0001. Values were determined by two‐tailed unpaired Student's t test (A, G and H), one‐way ANOVA followed by Bonferroni's post hoc test (A, J and K). Original blots can be found in Figure S7.

To investigate the clinical relevance of TOLLIP in psoriasis, we first analyzed its mRNA expression levels in two public GEO datasets (GSE13355 and GSE30999), comparing lesional and non‐lesional skin from psoriatic patients with healthy control skin. Our analysis demonstrated a significant upregulation of *TOLLIP* expression in psoriatic lesions compared with both non‐lesional and healthy skin (Figure 1A). We subsequently analyzed single‐cell RNA‑sequencing data (GSE228421) derived from whole‑skin biopsies obtained from lesional and non‑lesional sites of five individuals with psoriasis. Compared with non‑lesional skin, *TOLLIP* expression was significantly elevated in keratinocytes from psoriatic lesions (Figure 1B,C and Figure S1A). Receiver operating characteristic (ROC) curve analysis showed area under the curve (AUC) values of 0.8092 (GSE13355) and 0.7408 (GSE30999) (Figure 1D), indicating that TOLLIP can effectively distinguish psoriatic lesions from healthy skin. Consistent with these findings, the expression of keratinocyte activation‐related genes, including *KRT16* (encoding keratin‐16), *S100A9* (encoding calgranulin B), *IL17A* (encoding interleukin‐17A), and *CXCL10* (encoding C‐X‐C motif chemokine 10), was markedly elevated and showed a strong positive correlation with TOLLIP expression (Figure 1E and Figure S1B). Further analysis of clinical data from psoriasis patients revealed that *TOLLIP* mRNA levels were significantly decreased following 0–12 weeks of etanercept (a TNF inhibitor) therapy or 15 days of brodalumab (an anti‐IL‐17 receptor antibody) treatment (Figure S1C).

To gain further insight into the correlation between TOLLIP and psoriasis progression, we collected skin tissue samples from both psoriatic lesions and normal control skin, and performed immunohistochemical analysis of TOLLIP on skin sections. TOLLIP levels were markedly increased in lesional keratinocytes compared with control skin (Figure 1F). We also established an imiquimod (IMQ)‐induced psoriasis‐like mouse model [25] and evaluated the expression levels of TOLLIP under both physiological and IMQ‐induced pathological conditions (Figure S1D–G). Quantitative real‐time PCR (qPCR) and western blot analysis of skin tissue homogenates revealed elevated TOLLIP expression in the IMQ‐treated group compared to the vaseline control group (Figure 1G,H). Immunohistochemical staining of dorsal skin sections confirmed that TOLLIP expression was predominantly increased in the epidermal layer of mice subjected to the IMQ‐induced psoriasis model (Figure 1I). Additionally, we examined TOLLIP expression in keratinocytes stimulated with C4, a cytokine cocktail (IL‐17A, IL‐22, TNF‐α, and OSM) known to mimic psoriasis by inducing keratinocyte proliferation and inflammatory mediator secretion [26, 27, 28, 29]. Both qPCR and western blot analyses demonstrated increased TOLLIP levels in C4‐treated keratinocytes (Figure 1J,K). Collectively, these results indicate that TOLLIP expression is significantly upregulated in psoriatic lesions and closely associated with psoriasis exacerbation, suggesting its potential role in psoriasis pathogenesis.

### 2.2 Keratinocyte‑Specific Tollip Protects Against IMQ‑Induced Psoriasis‑Like Phenotype in Mice

**FIGURE 2:**
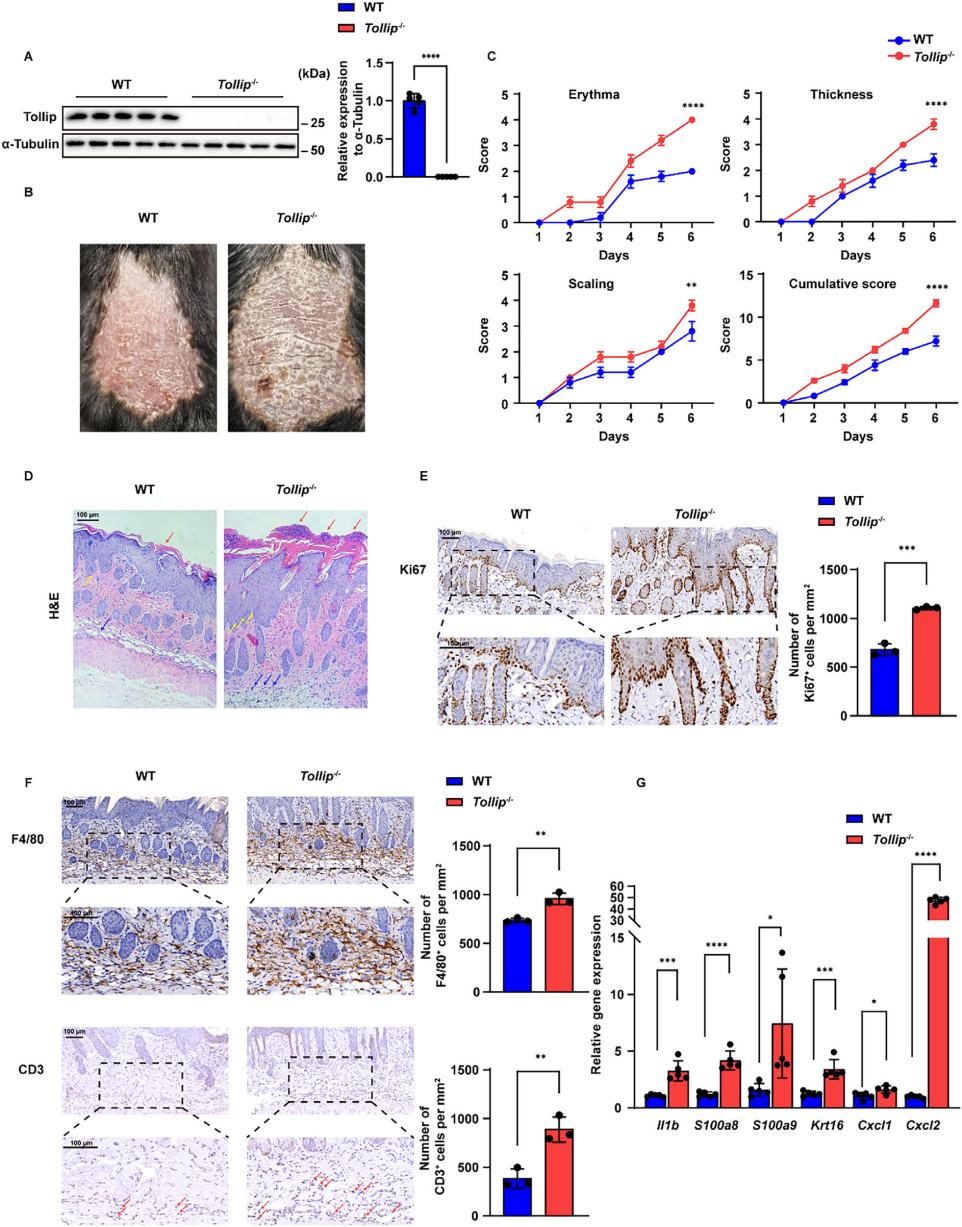
*Tollip* deficiency aggravates psoriasis‐like skin lesions. (A) Western blot analysis (left) and quantification (right) of Tollip protein expression in skin samples from wild‐type (WT) and *Tollip* knockout *(Tollip* ^−/−^) mice after 5 days of treatment with IMQ (*n* = 5 mice per group). α‐Tubulin was used as a loading control. (B) Representative gross appearance of shaved dorsal skin of WT and *Tollip* ^−/−^ mice after 5 days of treatment with IMQ (*n* = 5 mice per group). (C) Dorsal skin erythema, scaling, thickness, and their cumulative score of WT and *Tollip* ^−/−^ mice were scored daily based on the PASI scoring system with 5 days of treatment of IMQ (*n* = 5 mice per group). (D) Representative H&E staining images of dorsal skin sections from WT and *Tollip* ^−/−^ mice subjected to IMQ (*n* = 3 mice per group). The red arrows represented thickened stratum spinosum and stratum corneum, blue arrows represented inflammatory cell infiltrate, and yellow arrows represented subcutaneous hemorrhage. Scale bar, 100 µm. (E, F) Representative immunohistochemistry staining images (left) and quantitative analysis (right) showing Ki67‐positive (E), F4/80‐ and CD3‐positive (F) cells in dorsal skin sections from WT and *Tollip* ^−/−^ mice following 5‐day treatment with IMQ (*n* = 3 mice per group). Scale bar, 100 µm. (G) qPCR analysis of *Il1b*, *S100a8*, *S100a9*, *Krt16*, *Cxcl1*, and *Cxcl2* mRNA expression in skin samples from WT and *Tollip* ^−/−^ mice following 5‐day treatment with IMQ (*n* = 5 mice per group). *Tuba1a* was used as a reference gene for normalization. Data are shown as mean ± SD. ^*^ *p* < 0.05, ^**^ *p* < 0.01, ^***^ *p* < 0.001, ^****^ *p* < 0.0001. Values were determined by two‐way ANOVA followed by Bonferroni's post hoc test (C), two‐tailed unpaired Student's *t* test (E, F and G). Original blots can be found in Figure S8.

To investigate the role of TOLLIP in psoriasis pathogenesis, we first generated *Tollip* knockout (*Tollip* ^−/−^) mice using CRISPR‐Cas9 technology (Figure S2A). Genotyping was performed by PCR amplification of tail DNA, followed by agarose gel electrophoresis. The wild‐type (WT) allele produced a 4204‐bp band, whereas the knockout allele yielded a 580‐bp fragment due to targeted deletion (Figure S2B). Western blot analysis confirmed the complete absence of Tollip protein in *Tollip* ^−/−^ mice, while robust expression was detected in WT controls (Figure 2A). Next, we treated both WT and *Tollip* ^−/−^ mice with vaseline or IMQ cream on the shaved back daily for 5 days. In the vaseline‐treated groups, there was no difference between WT and *Tollip* ^−/−^ mice (Figure S2C–E). In contrast, following IMQ treatment, *Tollip* ^−/−^ mice exhibited significantly exacerbated psoriatic erythema, scaling, and epidermal thickening compared to WT mice (Figure 2B). Consistently, the Psoriasis Area and Severity Index (PASI) score was markedly elevated in *Tollip* ^−/−^ mice (Figure 2C). Histological analysis of hematoxylin and eosin (H&E)‐stained dorsal skin sections revealed pronounced epidermal hyperplasia, subcutaneous hemorrhage, and inflammatory infiltration in IMQ‐treated *Tollip* ^−/−^ mice (Figure 2D). Furthermore, immunohistochemical staining demonstrated a significant increase in Ki67^+^ proliferating keratinocytes in the epidermis of *Tollip* ^−/−^ mice (Figure 2E), suggesting enhanced epidermal hyperproliferation. We also observed elevated infiltration of CD3^+^ immune cells and macrophages (F4/80^+^) in the skin of *Tollip* ^−/−^ mice following IMQ treatment (Figure 2F). Correspondingly, qPCR analysis revealed upregulated mRNA expression of psoriasis‐associated genes, including inflammatory factors and chemokines (*Il1b*, *Cxcl1*, and *Cxcl2*), as well as the typical markers of keratinocyte activation and proliferation (*S100a8*, *S100a9*, and *Krt16*), in *Tollip* ^−/−^ mice compared with WT controls (Figure 2G). Taken together, these findings demonstrate that *Tollip* deficiency exacerbates IMQ‐induced psoriasis‐like phenotype, highlighting a critical protective role of TOLLIP in suppressing psoriasis development.

**FIGURE 3:**
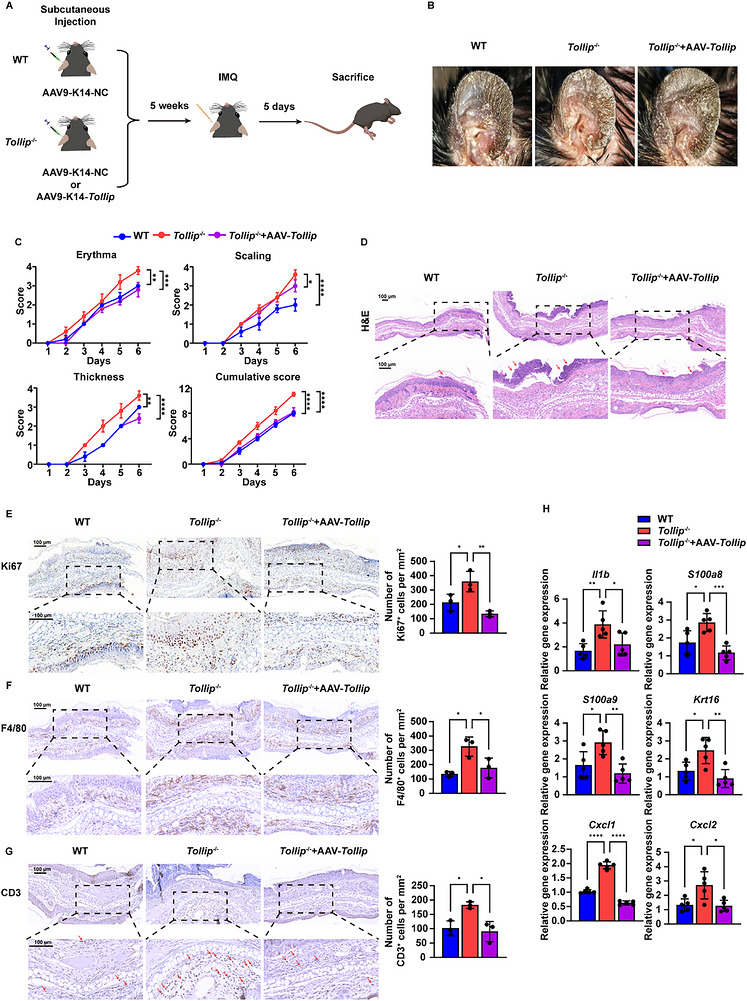
Keratinocyte‑specific re‑expression of *Tollip* ameliorates the psoriasis‑like skin lesions induced by *Tollip* deficiency. (A) Schematic diagram of the experimental design for subcutaneous injection of AAV9 vectors carrying either a negative control (NC) or *Tollip* into the ear skin of WT or *Tollip* ^−/−^ mice. AAV‐mediated gene expression was allowed to proceed for 5 weeks, followed by the construction of an IMQ‐induced psoriasis‐like mouse model. (B) Representative gross appearance of ears sections from WT, *Tollip* ^−/−^ and *Tollip* ^−/−^+AAV‐*Tollip* mice after 5 days of treatment with IMQ (*n* = 5 mice per group). (C) Ear erythema, scaling, thickness, and their cumulative score of WT, *Tollip* ^−/−^ and *Tollip* ^−/−^ *+*AAV‐*Tollip* mice were scored daily based on the PASI scoring system with 5 days of treatment of IMQ (*n* = 5 mice per group). (D) Representative H&E staining images of ear sections from WT, *Tollip* ^−/−^ and *Tollip* ^−/−^ *+*AAV‐*Tollip* mice subjected to IMQ (*n* = 3 mice per group). The red arrows represent thickened stratum corneum. Scale bar, 100 µm. (E, F and G) Representative immunohistochemistry staining images (left) and quantitative analysis (right) showing Ki67‐positive (E), F4/80‐positive (F), and CD3‐positive (G) cells in ear sections from WT, *Tollip* ^−/−^ and *Tollip* ^−/−^+AAV‐*Tollip* mice following 5‐day treatment with IMQ (n = 3 mice per group). Scale bar, 100 µm. (H) qPCR analysis of *Il1b*, *S100a8*, *S100a9*, *Krt16*, *Cxcl1* and *Cxcl2* mRNA expression in ear samples from WT, *Tollip* ^−/−^ and *Tollip* ^−/−^+AAV‐*Tollip* mice following 5‐day treatment with IMQ (*n* = 5 mice per group). *Tuba1a* was used as a reference gene for normalization. Data are shown as mean ± SD. ^*^ *p* < 0.05, ^**^ *p* < 0.01, ^***^ *p* < 0.001, ^****^ *p* < 0.0001. Values were determined by one‐way ANOVA followed by Bonferroni's post hoc test (E, F, G and H), two‐way ANOVA followed by Bonferroni's post hoc test (C).

To further investigate the role of keratinocyte‐specific Tollip in psoriasis progression, we performed rescue experiments by intradermally injecting adeno‐associated virus serotype 9 encoding *Tollip* under the control of the keratin K14 promoter (AAV9‐ K14‐*Tollip*) into *Tollip*‐KO mice, aiming to determine whether keratinocyte‐specific re‐expression of Tollip could ameliorate the exacerbated psoriatic phenotype in *Tollip*‐KO mice (Figure 3A and Figure S3). The results showed that re‐expression of Tollip in keratinocytes (*Tollip* ^−/−^+AAV‐*Tollip*) significantly alleviated the psoriatic lesions in IMQ‐induced *Tollip* ^−/−^ mice, as evidenced by a reduction of erythema, scaling, and PASI score (Figure 3B,C). H&E staining revealed a decrease in epidermal thickness in *Tollip* ^−/−^+AAV‐*Tollip* mice treated with IMQ, compared to that in *Tollip* ^−/−^ mice (Figure 3D). Furthermore, immunohistochemical analysis revealed a significant reduction in the expression of Ki67 in the epidermis of IMQ‐treated *Tollip* ^−/−^+AAV‐*Tollip* mice (Figure 3E). The elevated infiltration of CD3^+^ immune cells and macrophages (F4/80^+^) (Figure 3F,G), as well as the upregulated mRNA expression of psoriasis‐associated genes (*Il1b*, *S100a8*, *S100a9*, *Krt16*, *Cxcl1* and *Cxcl2*) observed in IMQ‐treated *Tollip* ^−/−^ mice, were also reduced following AAV9‐ K14‐*Tollip* injection (Figure 3H). In summary, these findings demonstrate that keratinocyte‐specific TOLLIP protects against IMQ‑induced psoriasis‑like phenotype in mice.

### 2.3 TOLLIP Inhibits Proliferation and Inflammation of Keratinocytes

Next, we employed gain‐ and loss‐of‐function approaches in HaCaT keratinocytes to explore TOLLIP's function in a C4‐induced psoriasis‐like model (Figure S4A). Given that epidermal hyperproliferation represents a hallmark of psoriasis, we first examined the effects of TOLLIP overexpression and knockdown on cell viability in HaCaT keratinocytes. Notably, EdU assays showed that TOLLIP overexpression significantly inhibited HaCaT cell proliferation, whereas TOLLIP knockdown promoted it (Figure 4A,B). CCK‐8 assays yielded similar results (Figure S4B). Since the C4 cytokine mixture activates multiple signaling pathways, including MAPK and NF‐κB, and induces pro‐inflammatory mediators in keratinocytes, we next assessed these downstream effects. TOLLIP overexpression attenuated C4‐induced phosphorylation of p65, p38, ERK, and JNK (Figure 4C). Correspondingly, cells overexpressing TOLLIP showed markedly reduced expression of keratinocyte‐related genes (*KRT6*, *KRT16*, *KRT17*, *IL1B*, *IL6*, *IL23A*, *S100A7*, *S100A8*, *S100A9*, *CXCL8*, *TNF*, and *CAMP*) following C4 stimulation (Figure 4E). Conversely, TOLLIP deficiency exacerbated MAPK and NF‐κB pathway activation as well as inflammatory cytokine production (Figure 4D,F). To further validate these findings in a more physiological system, we isolated primary keratinocytes from WT and *Tollip*‐KO mice (Figure S4C). Following C4 stimulation, phosphorylation of p65, p38, Erk, and Jnk was increased in *Tollip*‐KO primary keratinocytes (Figure 4G). The expression of the same keratinocyte‐related genes (*Il1b*, *Il6*, *Il23a*, *Krt16*, *S100a8*, *S100a9*, *Cxcl1*, *Cxcl2* and *Camp*) was also elevated (Figure 4H). Together, these results demonstrate that TOLLIP acts as a critical regulator of both keratinocyte proliferation and inflammation, two central pathological features of psoriasis.

FIGURE 4TOLLIP inhibits proliferation and psoriasis‐like inflammation in keratinocytes. (A, B) Cell proliferation was measured by EdU assay in TOLLIP‐overexpressed (A), TOLLIP‐knockdown (B) HaCaT cells, along with their respective controls, treated with C4 for 0 and 72 h. Ctrl, control. (C, D) Western blot analysis (left) and quantification (right) of p‐p65, p65, p‐p38, p38, p‐ERK1/2, ERK1/2, p‐JNK and JNK protein expression in TOLLIP‐overexpressed (C), TOLLIP‐knockdown (D) HaCaT cells, along with their respective controls, treated with C4 for 0 and 24 h. β‐actin was used as the loading control. (E, F) qPCR analysis of *KRT6*, *KRT16*, *KRT17*, *IL1B*, *IL6*, *IL23A*, *S100A7*, *S100A8*, *S100A9*, *CXCL8*, *TNF*, *and CAMP* mRNA expression in TOLLIP‐overexpressed (E), TOLLIP‐knockdown (F) HaCaT cells, along with their respective controls, treated with C4 for 0 and 24 h. *ACTB* was used as a reference gene for normalization. (G) Western blot analysis (left) and quantification (right) of p‐p65, p65, p‐p38, p38, p‐Erk1/2, Erk1/2, p‐Jnk and Jnk protein expression in primary keratinocytes from WT and *Tollip* ^−/−^ mice, treated with C4 for 0 and 24 h. α‐Tubulin was used as the loading control. (H) qPCR analysis of *Il1b*, *Il6*, *Il23a*, *Krt16*, *S100a8*, *S100a9*, *Cxcl1*, *Cxcl2*, *and Camp* mRNA expression in primary keratinocytes from WT and *Tollip* ^−/−^ mice, treated with C4 for 0 and 24 h. *Tubula* was used as a reference gene for normalization. Data represent three independent experiments and are shown as mean ± SD. ^*^ *p* < 0.05, ^**^ *p* < 0.01, ^***^ *p* < 0.001, ^****^ *p* < 0.0001. Values were determined by two‐way ANOVA followed by Bonferroni's post hoc test (A‐H). Original blots can be found in Figure S9.

**Figure:**
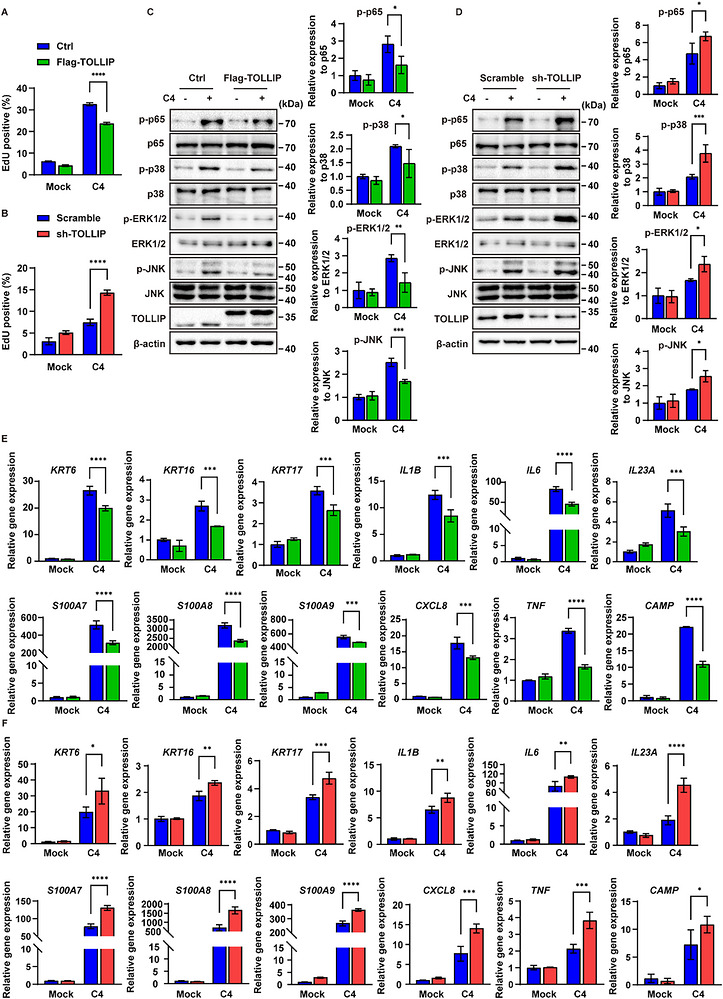

**Figure:**
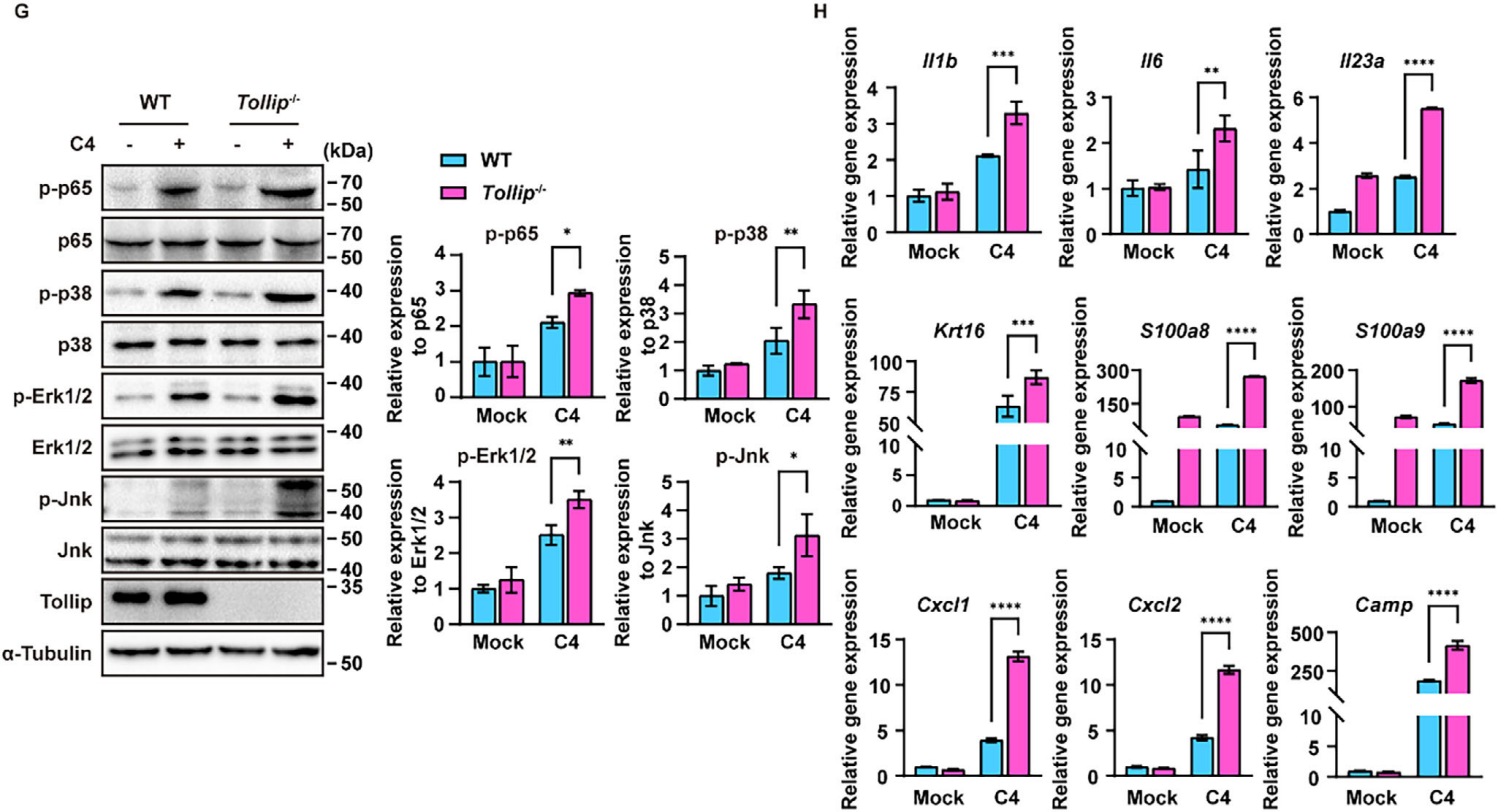

### 2.4 TOLLIP Interacts With PKM2 and Inhibits Its Activity

**FIGURE 5:**
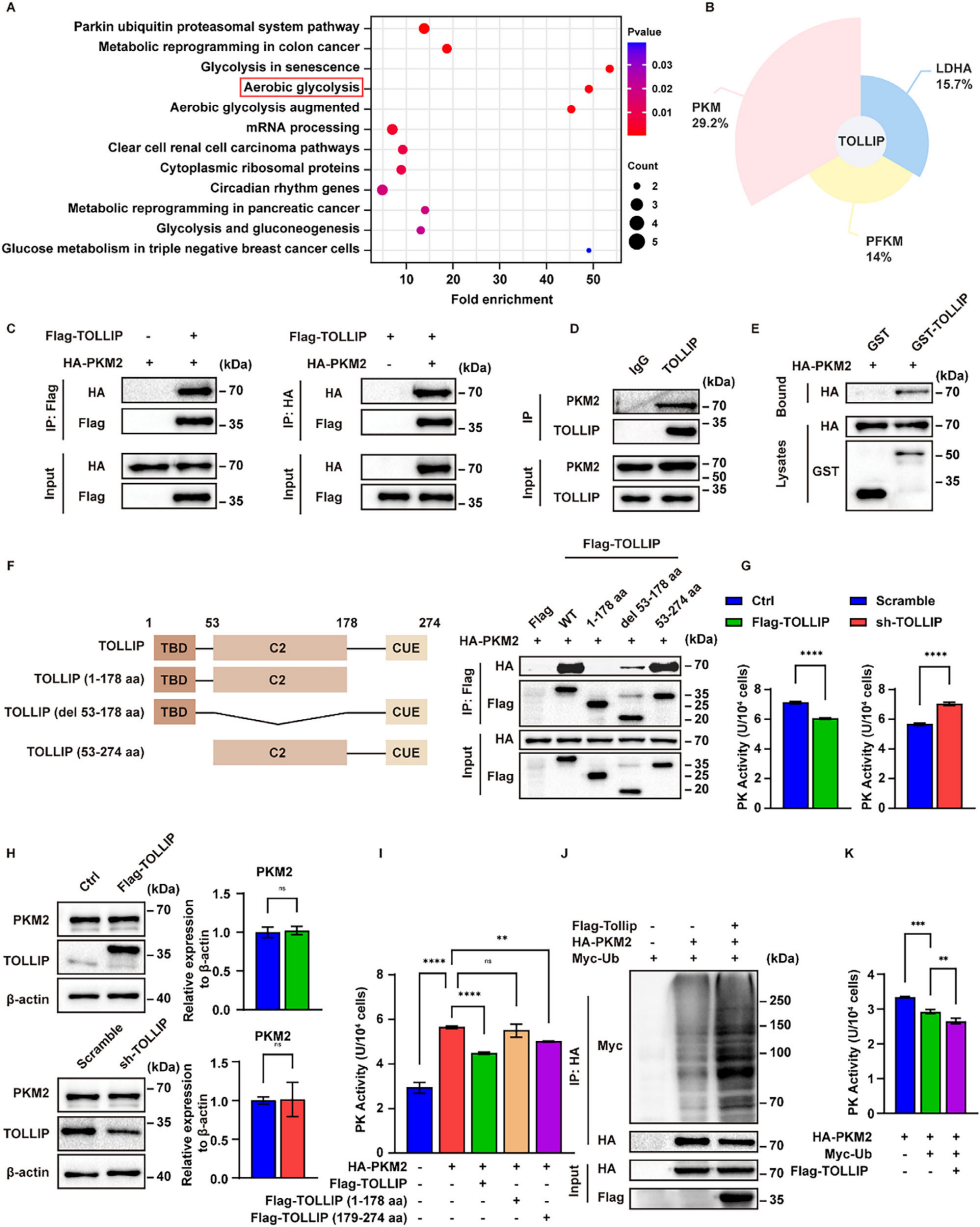
TOLLIP interacts with PKM2 and inhibits PKM2's activity. (A) Major biological pathways contributing to TOLLIP function determined by wikipathways enrichment analysis of mass spectrum from control and TOLLIP‐overexpressed cells. (B) Candidate interacting partners of TOLLIP within the glycolysis pathway. (C, D) Co‐immunoprecipitation analysis of TOLLIP and PKM2 interactions. Exogenous interaction following expression of Flag‐TOLLIP and HA‐PKM2 in HEK293T cells (C). Endogenous interaction in HaCaT cells (D). (E) GST‐pull‐down assay of TOLLIP and PKM2. Purified GST or GST‐TOLLIP protein was incubated with cell lysates expressing HA‐PKM2. Precipitated complexes were immunoblotted with the indicated antibodies. (F) Schematic representation of full‐length and truncated TOLLIP (left) and western blots for domain mapping analysis showing the binding domains of TOLLIP to PKM2 in HEK293T cells (right). (G) The effect of TOLLIP‐overexpression and ‐knockdown on pyruvate kinase activity. (H) Western blot analysis (left) and quantification (right) of PKM2 expression in TOLLIP‐overexpressed (upper), TOLLIP‐knockdown (lower) HaCaT cells, along with their respective controls. β‐actin was used as the loading control. (I) Identification of the key domain in TOLLIP essential for pyruvate kinase activation. (J) Immunoprecipitation assay showing the polyubiquitination of PKM2 in cells transfected with the indicated plasmids. Cell lysates were immunoprecipitated with anti‐HA antibody. (K) The effect of TOLLIP on the polyubiquitination‐mediated inhibition of PKM2 activity. Data represent three independent experiments and are shown as mean ± SD. ns, no significance, ^**^ *p* < 0.01, ***p < 0.001, ^****^ *p* < 0.0001. Values were determined by two‐tailed unpaired Student's *t*‐test (G, H) and one‐way ANOVA followed by Bonferroni's post hoc test (I, K). Original blots can be found in Figure S10.

To elucidate the molecular mechanisms by which TOLLIP influences psoriasis pathogenesis, we analyzed TOLLIP‐associated mass spectrometry data from our previous study [21]. Wikipathways analysis revealed aerobic glycolysis as a key metabolic pathway linked to TOLLIP function (Figure 5A). Among the glycolysis‐related enzymes identified in the TOLLIP interactome, pyruvate kinase M (PKM) was notable for its higher peptide coverage (Figure 5B). Furthermore, the expression level of TOLLIP showed a strong positive correlation with PKM in both healthy and psoriatic human skin samples (Figure S5A). Given the established role of PKM2 in psoriasis progression and its potential as a therapeutic target [11, 16], we focused subsequent studies on clarifying the functional relationship between TOLLIP and PKM2.

Co‐immunoprecipitation (Co‐IP) assays confirmed a robust exogenous interaction between TOLLIP and PKM2 (Figure 5C), which was further validated under endogenous conditions (Figure 5D). Supporting these findings, an in vitro GST pull‐down assay demonstrated that purified GST‐TOLLIP could associate with PKM2 (Figure 5E and Figure S5B). Domain mapping experiments revealed that the CUE domain (179–274 aa) of TOLLIP is essential for this interaction, as its deletion abrogated binding to PKM2 (Figure 5F). Functional characterization showed that TOLLIP significantly suppressed PKM2 enzymatic activity without altering its protein abundance (Figure 5G,H). The CUE domian (179–274 aa) was identified as the primary functional domain responsible for inhibiting PKM2's activity, with no effect on PKM2 expression (Figure 5I and Figure S5C). Since ubiquitination has been shown to inhibit PKM2 activity [30] and the CUE domain of TOLLIP binds ubiquitinated proteins [31, 32], we hypothesized that TOLLIP promotes PKM2 ubiquitination to suppress its enzymatic function. Consistent with this, experimental data confirmed that TOLLIP indeed enhances PKM2 ubiquitination, resulting in the inhibition of its activity (Figure 5J,K). Using a CUE domain mutant (MF/AA) of TOLLIP with impaired ubiquitin‐binding ability [33, 34], we examined its interaction with PKM2. The results showed that this mutation dramatically reduced the interaction between TOLLIP and PKM2, further supporting that TOLLIP recognizes PKM2 in a ubiquitin‐dependent manner (Figure S5D). Together, these results establish TOLLIP as a novel regulator of PKM2 that acts through its CUE domain (179–274 aa) to inhibit PKM2 enzymatic activity, suggesting a potential mechanism by which TOLLIP may influence glycolytic reprogramming in psoriatic phenotypes.

### 2.5 TOLLIP Attenuates Psoriasis‐Like Phenotypes by Suppressing PKM2‐Mediated Glycolysis in Keratinocytes

**FIGURE 6:**
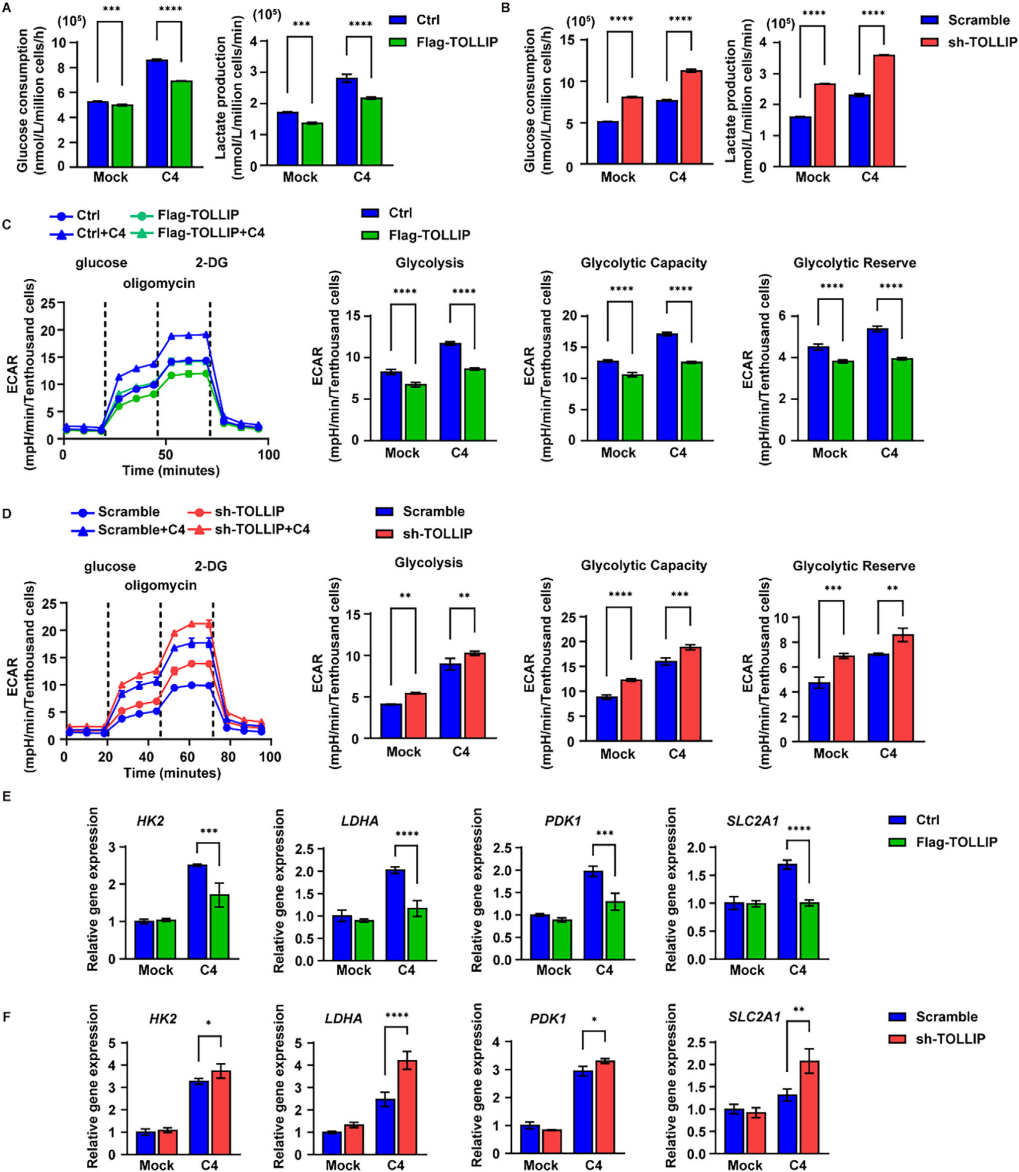
TOLLIP inhibits aerobic glycolysis in keratinocytes. (A, B) Glucose consumption (left) and lactate production (right) were measured in TOLLIP‐overexpressed (A), TOLLIP‐knockdown (B) HaCaT cells, along with their respective controls, treated with C4 for 0 and 24 h. (C, D) Extracellular acidification rate (ECAR) assay in TOLLIP‐overexpressed (C), TOLLIP‐knockdown (D) HaCaT cells, along with their respective controls, treated with C4 for 0 and 24 h. (E, F) qPCR analysis of *HK2*, *LDHA*, *PDK1* and *SLC2A1* mRNA expression in TOLLIP‐overexpressed (E), TOLLIP‐knockdown (F) HaCaT cells, along with their respective controls, treated with C4 for 0 and 24 h. *ACTB* was used as a reference gene for normalization. Data represent three independent experiments and are shown as mean ± SD. ^*^ *p* < 0.05, ^**^ *p* < 0.01, ^***^ *p* < 0.001, ^****^ *p* < 0.0001. Values were determined by two‐way ANOVA followed by Bonferroni's post hoc test (A–F).

We further investigated the impact of TOLLIP on aerobic glycolysis in keratinocytes using a psoriasis‐like model induced by C4 stimulation. The results showed that overexpression of TOLLIP significantly suppressed glucose consumption, lactate production and extracellular acidification rate (ECAR), a proxy for glycolytic rate and capacity, in C4‐stimulated HaCaT cells (Figure 6A,C). Additionally, transcriptional expression levels of hexokinase 2 (*HK2*), lactate dehydrogenase A (*LDHA*), pyruvate dehydrogenase kinase 1 (*PDK1*) and solute carrier family 2 member 1 (*SLC2A1*) were notably decreased in HaCaT cells overexpressing TOLLIP (Figure 6E). Conversely, TOLLIP deficiency enhanced glycolytic capacity in HaCaT cells (Figure 6B,D,F). These findings demonstrate TOLLIP's capacity to inhibit aerobic glycolysis in keratinocytes.

**FIGURE 7:**
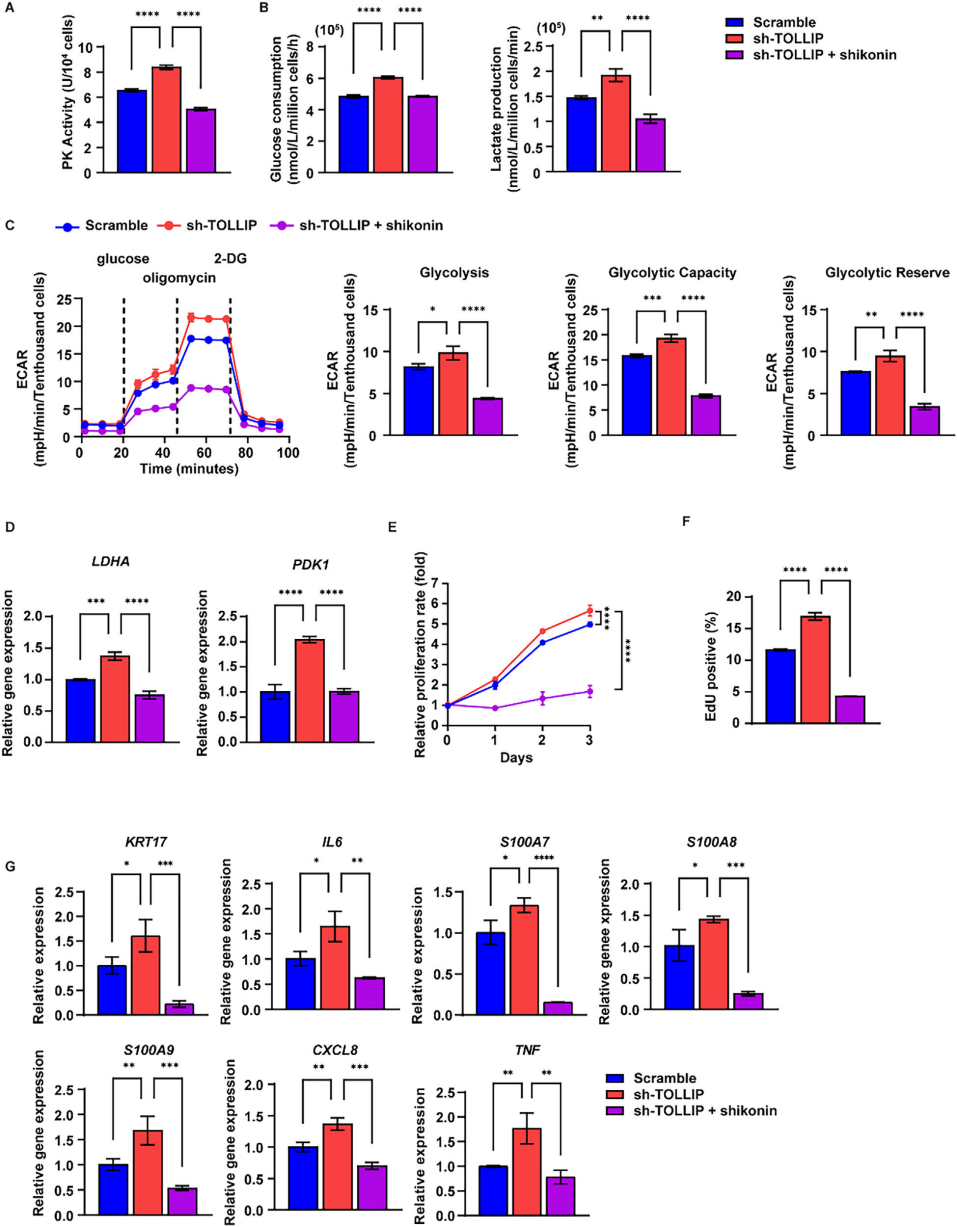
Shikonin, a PKM2 inhibitor, ameliorates severe psoriasis‐like phenotypes induced by TOLLIP knockdown. (A) Pyruvate kinase activity was measured in scramble control cells, TOLLIP‐knockdown cells, and TOLLIP‐knockdown cells treated with shikonin, after 24 h of treatment with C4. (B) Glucose consumption (left) and lactate production (right) assay in scramble control cells, TOLLIP‐knockdown cells, and TOLLIP‐knockdown cells treated with shikonin, after 24 h of treatment with C4. (C) Extracellular acidification rate assay in scramble control cells, TOLLIP‐knockdown cells, and TOLLIP‐knockdown cells supplemented with shikonin, after 24 h of treatment with C4. (D) qPCR analysis of *LDHA* and *PDK1* mRNA expression in scramble control cells, TOLLIP‐knockdown cells, and TOLLIP‐knockdown cells supplemented with shikonin, after 24 h of treatment with C4. *ACTB* was used as a reference gene for normalization. (E) Proliferation curves of the scramble control cells, TOLLIP‐knockdown cells, and TOLLIP‐knockdown cells supplemented with shikonin, after indicated times of treatment with C4. (F) EdU assay for cell proliferation in scramble control cells, TOLLIP‐knockdown cells, and TOLLIP‐knockdown cells treated with shikonin at 72 h after C4 stimulation. (G) qPCR analysis of *KRT17*, *IL6*, *S100A7*, *S100A8*, *S100A9*, *CXCL8* and *TNF* mRNA expression in scramble control cells, TOLLIP‐knockdown cells, and TOLLIP‐knockdown cells supplemented with shikonin, after 24 h of treatment with C4. *ACTB* was used as a reference gene for normalization. Data represent three independent experiments and are shown as mean ± SD. ^*^ *p* < 0.05, ^**^ *p* < 0.01, ^***^ *p* < 0.001, ^****^ *p* < 0.0001. Values were determined by one‐way ANOVA followed by Bonferroni's post hoc test (A–D, F and G), two‐way ANOVA followed by Bonferroni's post hoc test (E).

Shikonin, a major chemical component derived from the roots of Lithospermum erythrorhizon (Purple Cromwell), acts as a specific inhibitor of PKM2 [35, 36]. We found that shikonin effectively counteracted the increase in PKM2 activity induced by TOLLIP knockdown in HaCaT cells (Figure 7A). Consistently, we observed that the elevated glucose consumption, lactate production, ECAR, and the expression levels of *LDHA* and *PDK1* expression resulting from TOLLIP deficiency in HaCaT cells were significantly antagonized by shikonin treatment (Figure 7B–D). Furthermore, functional assays showed that shikonin could reverse the increased proliferation and the upregulated expression of psoriasis‐associated markers (*KRT17*, *IL6*, *S100A7*, *S100A8*, *S100A9*, *CXCL8* and *TNF*) caused by TOLLIP‐deficiency (Figure 7E–G). These findings indicated that TOLLIP suppresses psoriasis‐like phenotypes, including keratinocytes proliferation and inflammation by repressing PKM2 activity‐dependent aerobic glycolysis.

### 2.6 TOLLIP (179–274 aa) Is Required for Suppressing Aerobic Glycolysis and Psoriasis‐Like Phenotypes

**FIGURE 8:**
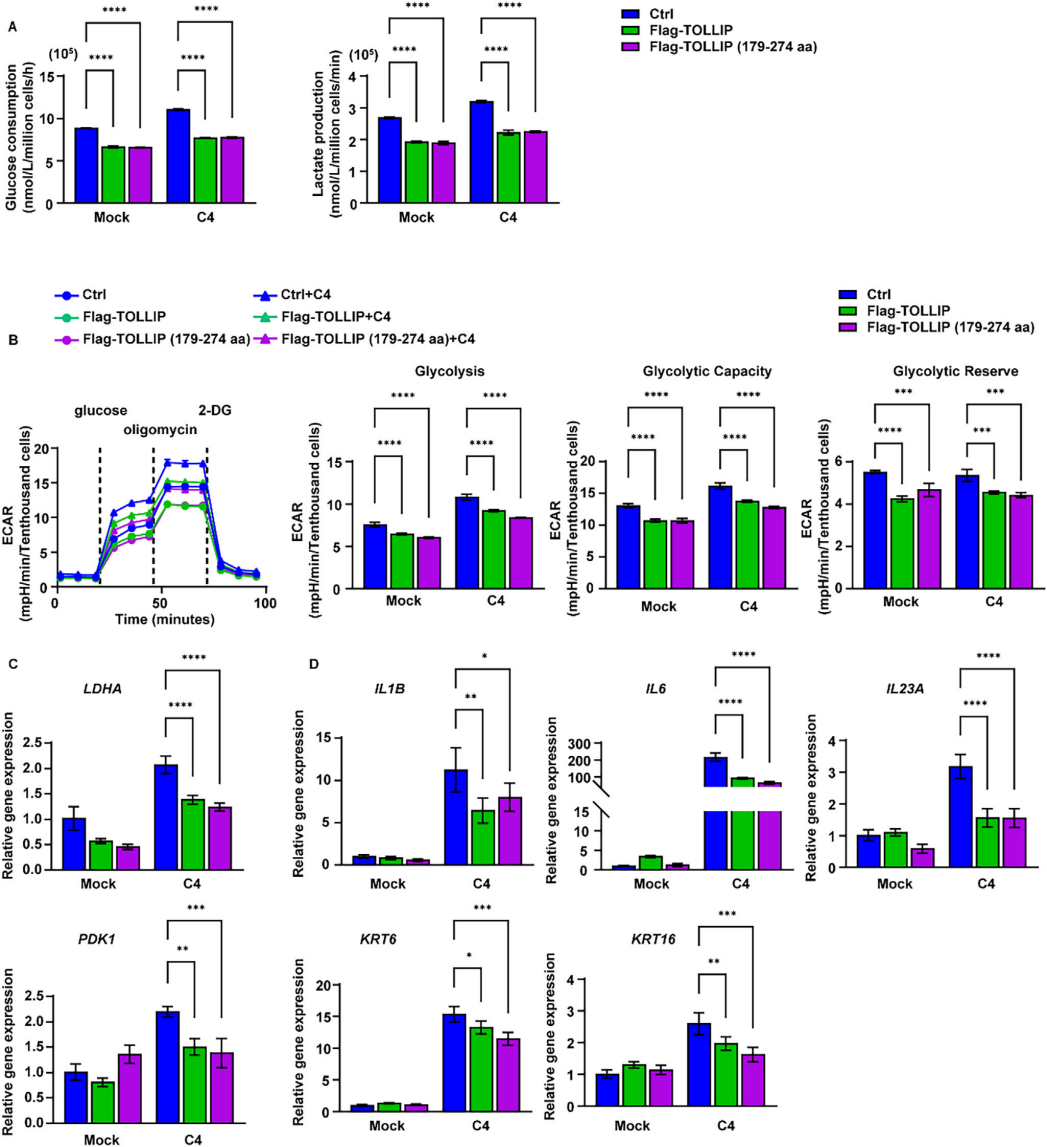
TOLLIP (179‐274 aa) inhibits aerobic glycolysis, proliferation and inflammation of keratinocytes. (A) Glucose consumption (left) and lactate production (right) were measured in control, TOLLIP‐ and TOLLIP (179–274 aa)‐overexpressed HaCaT cells after treatment with C4 for 0 and 24 h. (B) Extracellular acidification rate assay in control, TOLLIP‐ and TOLLIP (179‐274 aa)‐overexpressed HaCaT cells after 0 and 24 h treatment with C4. (C) qPCR analysis of *LDHA* and *PDK1* mRNA expression in control, TOLLIP‐ and TOLLIP (179–274 aa)‐overexpressed HaCaT cells after 0 and 24 h treatment with C4. *ACTB* was used as a reference gene for normalization. (D) qPCR analysis of *IL1B*, *IL6*, *IL23A*, *KRT6* and *KRT16* mRNA expression in control, TOLLIP‐ and TOLLIP (179–274 aa)‐overexpressed HaCaT cells after 0 and 24 h treatment with C4. *ACTB* was used as a reference gene for normalization. Data represent three independent experiments and are shown as mean ± SD. ^*^ *p* < 0.05, ^**^ *p* < 0.01, ^***^ *p* < 0.001, ^****^ *p* < 0.0001. Values were determined by two‐ way ANOVA followed by Bonferroni's post hoc test (A–D).

Given the established role of the TOLLIP (179‐274 aa) in regulating PKM2 activity (Figure 5I), we sought to determine whether this domain contributes to the modulation of aerobic glycolysis and inflammation in psoriatic keratinocytes. Strikingly, TOLLIP (179–274 aa) was sufficient to suppress glucose consumption, lactate production, and ECAR to a degree comparable to that of full‐length TOLLIP (Figure 8A,B). Furthermore, the expression levels of glycolysis‐related genes (*LDHA, PDK1*) and psoriasis‐associated genes (*IL1B*, *IL6*, *IL23A*, *KRT6*, *KRT16*) were significantly reduced in HaCaT cells expressing TOLLIP (179–274 aa) (Figure 8C,D). These findings indicate that TOLLIP (179‐274 aa) is required for inhibiting glycolysis and psoriasis‐like phenotypes in keratinocytes.

To investigate the keratinocyte‐specific role of TOLLIP and TOLLIP (179–274 aa) in psoriasis pathogenesis, we delivered AAV9 driven by the K14 promoter to IMQ‐induced psoriatic mice. Mice were randomly assigned to three groups receiving AAV‐*Tollip*, AAV‐*Tollip* (179–274 aa), or AAV‐NC, respectively (Figure 9A). Five weeks after subcutaneous AAV administration, both AAV‐*Tollip* and AAV‐*Tollip* (179‐274 aa) led to substantially increased levels of their respective exogenous proteins in the epidermis of the mouse dorsal skin (Figure S6A,B). Following IMQ application, both AAV‐*Tollip* and AAV‐*Tollip* (179‐274 aa) treatments markedly ameliorated key psoriatic features, including erythema, scaling, epidermal hyperplasia, and Ki67^+^ keratinocyte proliferation (Figure 9B–E). Additionally, this treatment significantly decreased the infiltration of CD3^+^ immune cells and macrophages in the skin following IMQ treatment (Figure 9F). Consistent with these observations, the mRNA levels of aerobic glycolysis‐associated markers (*Pdk1* and *Slc2a1*) and psoriasis‐associated markers (*Krt16*, *Il23a*, *Cxcl1*, *Cxcl2*, *S100a8* and *S100a9*) were substantially downregulated in AAV‐*Tollip* and AAV‐*Tollip* (179–274 aa)‐treated mice (Figure 9G,H). Collectively, these data highlight the indispensable role of the TOLLIP C‐terminal region (179–274 aa) in mitigating aerobic glycolysis and psoriasis‐like phenotypes, both in keratinocytes and in vivo, reinforcing its potential as a therapeutic target in psoriasis.

FIGURE 9Tollip and its C‐terminal domain (179–274 aa) inhibit psoriasis‐like skin lesions. (A) Schematic diagram of the experimental design for subcutaneous injection of AAV9 vectors carrying either a negative control (NC), *Tollip*, or *Tollip* (179–274 aa) into the dorsal skin of mice. Transgene expression was allowed to proceed for 5 weeks, followed by the construction of an IMQ‐induced psoriasis‐like mouse model to investigate the therapeutic potential of the expressed genes. (B) Representative gross appearance of shaved dorsal skin from mice subcutaneously injected with AAV9 vectors. Mice were injected with AAV9 carrying NC, *Tollip*, or *Tollip* (179–274 aa), followed by 5‐day topical treatment with control cream or IMQ (*n* = 5 mice per group). (C) Representative H&E staining images of dorsal skin sections from mice subcutaneous injected with AAV9 vectors carrying NC, *Tollip*, and *Tollip* (179‐274 aa), followed by 5‐day topical treatment with control cream or IMQ (*n* = 3 mice per group). The red arrows represented thickened stratum spinosum and stratum corneum, blue arrows represented inflammatory cell infiltrate, and yellow arrows represented sub cutaneous hemorrhage. Scale bar, 100 µm. (D) Dorsal skin erythema, scaling, and thickness of NC, Tollip‐ and Tollip (179–274 aa)‐overexpressed mice were scored daily based on the PASI scoring system with 5 days of treatment with control cream or IMQ (*n* = 5 mice per group). (E, F) Representative immunohistochemistry staining images (left) and quantitative analysis (right) showing Ki‐67‐positive (E), F4/80‐positive and CD3‐positive (F) cells in dorsal skin sections from NC, Tollip‐ and Tollip (179–274 aa)‐overexpressed mice after 5 days of treatment with control cream or IMQ (*n* = 3 mice per group). Scale bar, 100 µm. (G, H) qPCR analysis of *Pdk1*, *Slc2a1* (G), *Krt16*, *Il23a*, *Cxcl1*, *Cxcl2*, *S100a8* and *S100a9* (H) mRNA expression in mice subcutaneous injected with AAV9 vectors carrying NC, *Tollip* and *Tollip* (179–274 aa), followed by 5‐day topical treatment with control cream or IMQ. *Tuba1a* was used as a reference gene for normalization (*n* = 5 mice per group). Data are shown as mean ± SD. ^*^ *p* < 0.05, ^**^ *p* < 0.01, ^***^ *p* < 0.001, ^****^ *p* < 0.0001. Values were determined by one‐way ANOVA followed by Bonferroni's post hoc test (E–H) and two‐way ANOVA followed by Bonferroni's post hoc test (D).

**Figure:**
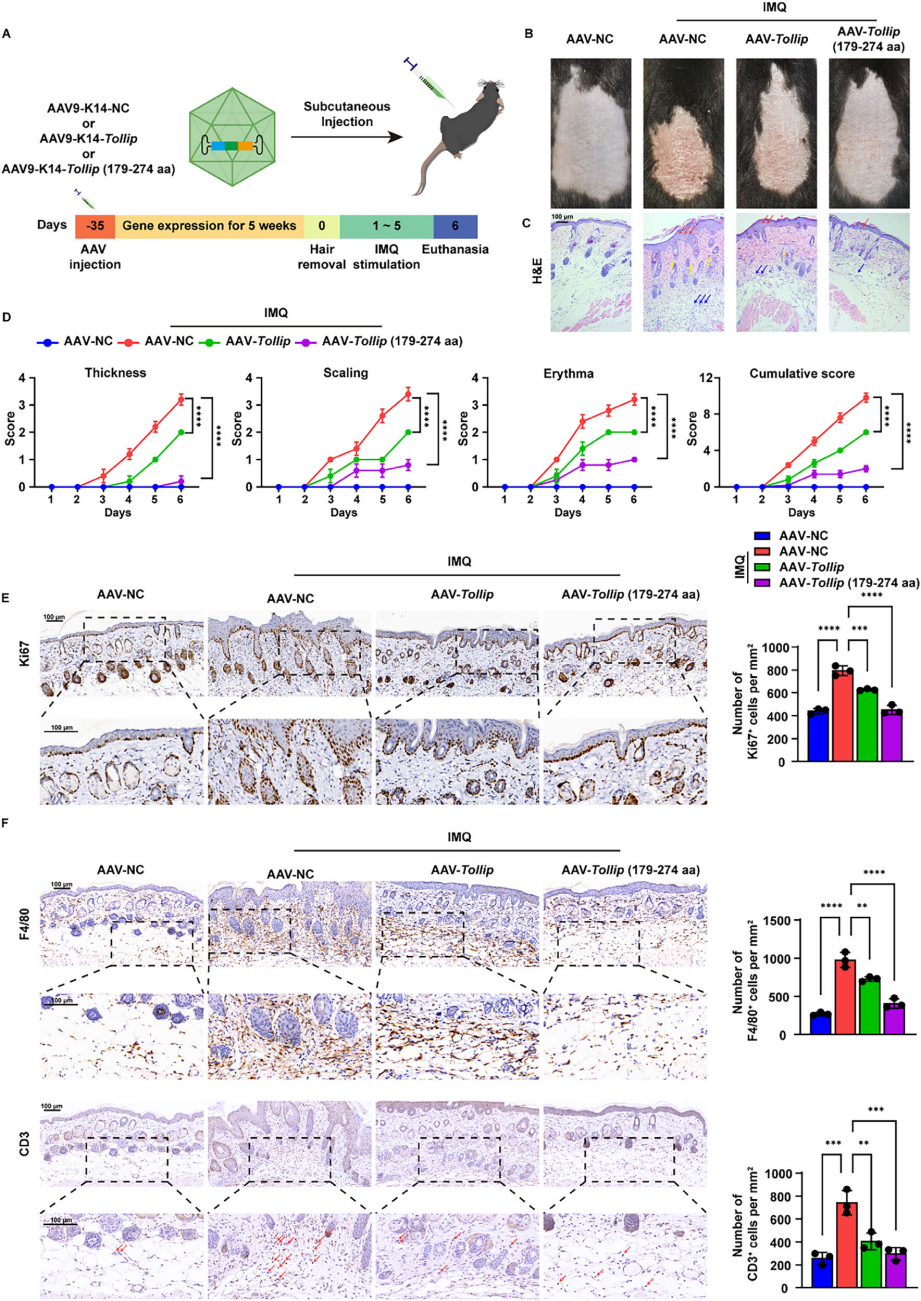

**Figure:**
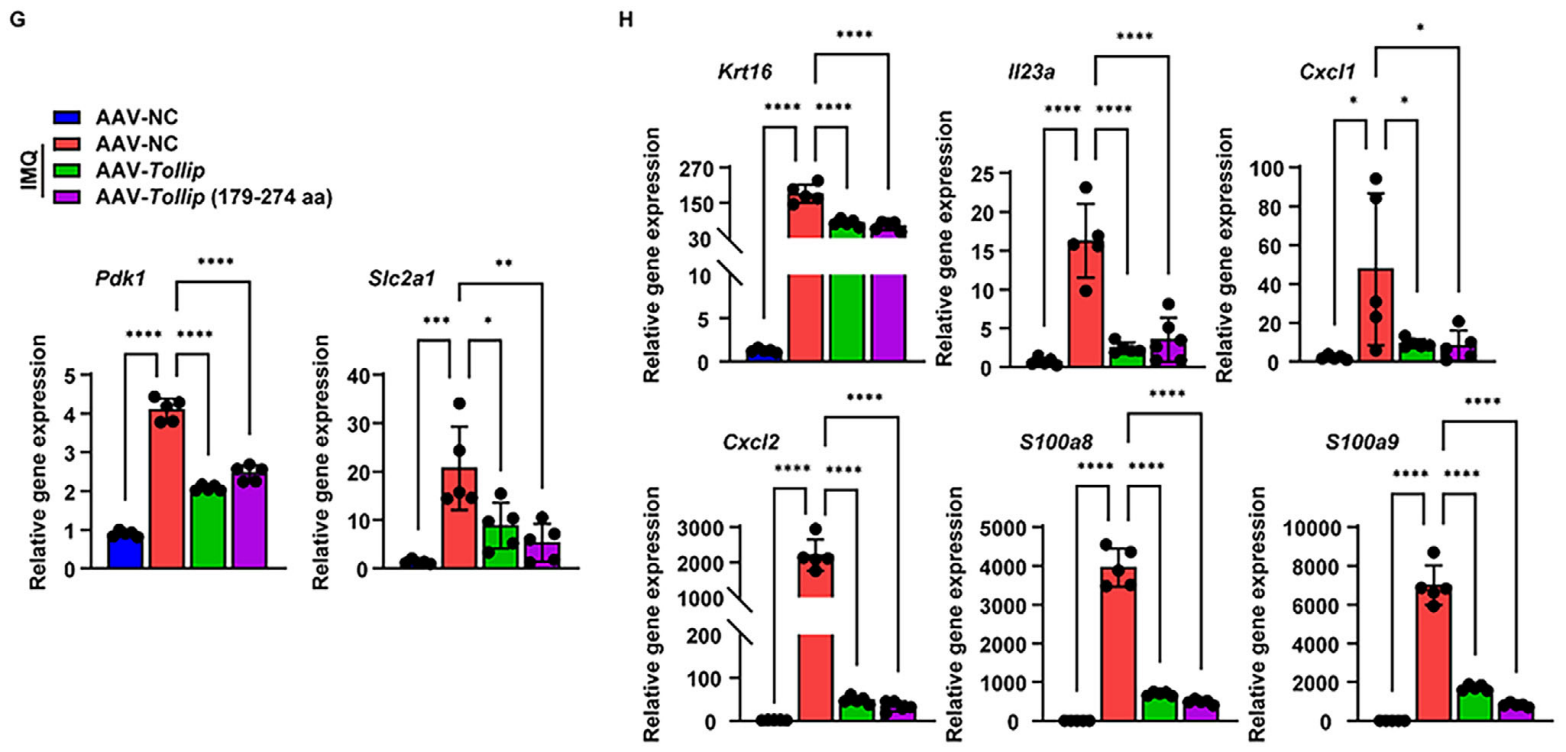

## 3 Discussion

**FIGURE 10:**
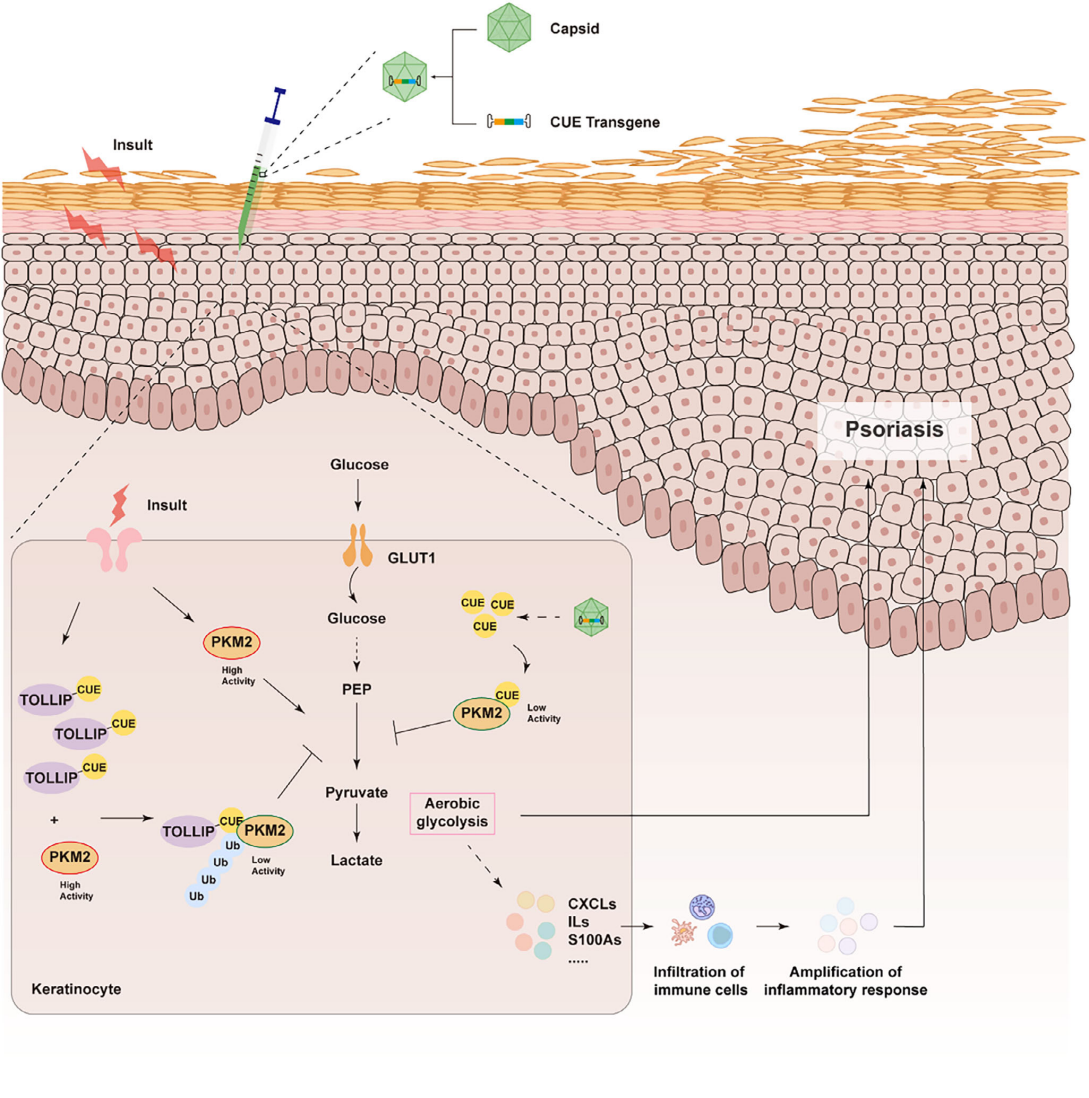
Schematic diagram illustrating the role of TOLLIP and its CUE truncation mutant (179–274 aa) in alleviating psoriasis progression. Upon skin lesion formation, upregulated TOLLIP interacts with PKM2 and inhibits its activity by promoting PKM2's polyubiquitination. This suppression attenuates aerobic glycolysis, thereby reducing hyperproliferation and inflammation of keratinocytes. In therapeutic settings, AAV‐mediated delivery of either full‐length TOLLIP or its CUE truncation mutant alone is sufficient to ameliorate psoriasis‐like symptoms in mouse models, highlighting the potential of TOLLIP‐based strategies for psoriasis treatment. PEP, phosphoenolpyruvate.

The multifaceted roles of TOLLIP as an intracellular adaptor in innate immunity and autophagy are well‐established [37, 38, 39]. However, a role for TOLLIP in keratinocyte metabolism during psoriasis pathogenesis has eluded discovery. Our study fundamentally expands this paradigm by revealing that TOLLIP acts as a critical metabolic brake in psoriasis through its direct binding to PKM2—a key regulator that controls glycolytic flux—thus inhibiting PKM2 enzymatic activity (Figure 10). This finding extends TOLLIP's function beyond its traditional roles in signaling and trafficking, raising a key question: what is the mechanism by which it achieves such potent control over a central metabolic pathway? The following discussion dissects the mechanistic and therapeutic implications of this newly discovered TOLLIP‐PKM2‐glycolysis axis.

Our in vivo and in vitro data collectively establish that TOLLIP exerts a cell‑autonomous protective function in keratinocytes. Specifically, we found that *Tollip* deficiency in primary mouse keratinocytes, independent of immune cell infiltration, is sufficient to drive hyperproliferation and inflammation, as evidenced by enhanced MAPK/NF‑κB activation and upregulation of psoriasis‑associated genes. This is consistent with the established role of keratinocytes as primary drivers of psoriatic epidermal hyperplasia [10]. Moreover, the concordant results from human HaCaT cells and primary mouse keratinocytes suggest that the protective function of TOLLIP is not cell‑line‑specific and likely reflects a conserved mechanism across species. These findings, together with our in vivo observations that keratinocyte‑specific TOLLIP overexpression suppresses IMQ‑induced psoriasis, strongly support a model in which TOLLIP acts as a cell‑autonomous brake on keratinocyte hyperproliferation and inflammation, positioning it as a key regulator of keratinocyte biology in psoriasis.

Despite its protective role, TOLLIP expression was markedly elevated in psoriasis patients compared to healthy controls—an observation that might initially appear paradoxical. However, our in vivo and in vitro studies provide direct functional evidence that TOLLIP is indeed protective. Notably, successful treatment with etanercept (a TNF inhibitor) or brodalumab (an anti‐IL‐17 receptor antibody) led to a significant reduction in TOLLIP levels. This dynamic expression pattern—upregulation during active disease and downregulation upon therapeutic intervention—suggests a compensatory protective response whereby the body attempts to counteract psoriatic pathogenesis. However, the persistent disease milieu (i.e., the sustained high concentrations of pro‑inflammatory cytokines in the psoriatic microenvironment) may continuously drive keratinocyte activation beyond TOLLIP's inhibitory capacity, limiting its therapeutic efficacy. Nevertheless, such paradoxical regulation is not unprecedented in the study of disease. For instance, SOD2 is upregulated in oxidative stress‐related disorders, such as Alzheimer's disease, to mitigate ROS‐induced damage [40]. Heat shock protein 27 exhibits compensatory overexpression in ischemia‐induced myocardial injury to provide cardioprotection against oxidative stress, apoptosis, and infarction [41]. These examples underscore the complexity of disease regulatory networks, where seemingly contradictory expression patterns, such as compensatory induction vs. functional inhibition, often reflect the body's adaptive responses. Importantly, functional validation—as performed in this study for TOLLIP—is essential to distinguish compensatory upregulation from pathogenic mechanisms.

Having established the protective role of TOLLIP in psoriasis, we next sought to define its mechanism of action. We discovered that TOLLIP exerts its anti‐psoriatic effects via direct engagement with cellular metabolism. Our data robustly demonstrate that TOLLIP, via its CUE domain, physically interacts with PKM2 and suppresses its enzymatic activity. This inhibition attenuates the aerobic glycolysis that fuels keratinocyte hyperproliferation and inflammation, positioning the TOLLIP‐PKM2 interaction as a central mechanism governing the metabolic network in psoriasis. Mechanistically, we found that TOLLIP inhibits PKM2 enzymatic activity by promoting PKM2 ubiquitination via its CUE domain, without affecting PKM2 protein stability. It is well established that the CUE domain functions as a ubiquitin‐binding domain rather than possessing E3 ligase activity [42]. Therefore, we propose that TOLLIP acts as a ubiquitin adaptor that recognizes pre‑existing ubiquitin chains on PKM2. This model is supported by the observation that a CUE domain mutant (MF/AA) with impaired ubiquitin‐binding ability exhibited a dramatically reduced interaction with PKM2, confirming that TOLLIP recognizes PKM2 in a ubiquitin‐dependent manner. This ubiquitin receptor function is consistent with the role of ubiquitin‐binding domains in promoting ubiquitination, either by stabilizing the interaction between substrates and E3 ligases or by protecting growing ubiquitin chains from premature deubiquitination [42]. The precise E3 ligase(s) involved in PKM2 ubiquitination and whether TOLLIP recruits or antagonizes specific enzymes remain to be identified in future studies.

Regarding the observation that increased ubiquitination does not lead to PKM2 degradation, this can be explained by the emerging concept of the ubiquitin code: different ubiquitin chain linkages confer distinct functional outcomes. While K48‐linked polyubiquitination typically targets proteins for proteasomal degradation, K63‐linked or other non‐degradative ubiquitin chains primarily regulate protein activity, subcellular localization, or protein–protein interactions without promoting degradation [43]. Our preliminary data suggest that TOLLIP may promote non‐degradative ubiquitination on PKM2, which could inhibit its enzymatic activity. Future studies using linkage‐specific ubiquitin antibodies or mass spectrometry‐based ubiquitinomics will be necessary to definitively determine the ubiquitin chain types on PKM2 and elucidate how these modifications allosterically regulate PKM2 activity.

A recent study has reported that PKM2 mediates IL‐17A signaling in keratinocytes by forming a complex with Act1 and TRAF6, thereby activating NF‐κB and driving psoriatic inflammation beyond its canonical metabolic activity [16]. This finding, together with our observation that TOLLIP inhibits PKM2 enzymatic activity through ubiquitination, raises the intriguing possibility that TOLLIP may regulate psoriatic progression through dual mechanisms involving PKM2. On one hand, TOLLIP directly suppresses PKM2's glycolytic function, as demonstrated in this study. On the other hand, TOLLIP might also disrupt the PKM2‐Act1‐TRAF6 complex formation, thereby attenuating IL‐17A‐induced inflammatory signaling. This would position TOLLIP as a multifaceted regulator that integrates metabolic control with immune modulation. However, whether TOLLIP directly affects PKM2's scaffolding function within the IL‐17A signaling complex remains to be elucidated and warrants further investigation.

While the scaffolding function remains to be fully defined, our demonstration that TOLLIP directly suppresses PKM2's glycolytic activity already provides a clear and targetable mechanism for therapeutic intervention. The therapeutic potential of targeting the TOLLIP‐PKM2 axis is particularly compelling in the context of keratinocytes, the primary drivers of psoriatic pathogenesis [10, 16, 44]. Our findings confirm that modulating glycolytic flux in keratinocytes is an effective strategy for curbing disease progression. The success of AAV‐mediated, keratinocyte‐specific *TOLLIP* gene delivery in mice underscores this potential. The pinpointing of a minimal 95‐amino‐acid functional domain within TOLLIP offers a direct path toward a novel class of targeted therapies through peptide‐based intervention. We propose that this domain could be engineered into a stable, cell‐permeable peptide drug, a strategy in line with the advancing field of peptide therapeutics [45]. Such an agent could offer a novel, mechanism‐based treatment option that directly addresses the metabolic core of psoriasis, potentially overcoming limitations of current biologics such as high cost and transient efficacy [46]. Nevertheless, the precise structural mechanism by which the CUE domain inhibits PKM2 activity remains to be elucidated. Revealing the atomic structure of this interface by co‐crystallography would address a key scientific question and guide the development of targeted drugs [47]. Furthermore, our work lays the foundation for translational engineering aimed at distilling the 95‐amino‐acid CUE fragment into an even smaller, pharmacologically optimized peptide candidate, a pursuit that holds promise for developing a first‐in‐class metabolic therapy for psoriasis.

Collectively, our study reveals a previously unrecognized function of TOLLIP in suppressing psoriasis progression through metabolic reprogramming. By modulating PKM2‐dependent aerobic glycolysis in keratinocytes, TOLLIP serves as a critical checkpoint in psoriatic inflammation and hyperproliferation. These findings position TOLLIP as a potential therapeutic target for developing novel anti‐psoriatic strategies.

## 4 Methods

### 4.1 Human Skin Samples

A total of 10 paraffin‐embedded sections were obtained, including 5 from patients with psoriasis (5 males, aged 10–36 years) and 5 from control subjects (1 male and 4 females, aged 9–52 years), for immunohistochemical analysis. Written informed consent was obtained from all participants. This study was performed in accordance with the Declaration of Helsinki principles and was approved by the Medical Ethics Committee of Hebei University of Engineering Affiliated Hospital (Approval No. 2026[K]024).

### 4.2 Animals

Wild‐type female C57BL/6 mice (6–8 weeks) were purchased from SPF (Beijing) Biotechnology Co., Ltd. *Tollip* ^−/−^ mice on the C57BL/6 background were provided by Shanghai southern Model Biotechnology Co., Ltd. Primer‐forward: 5’AGGGTCTGGTTTGCCTTGAG3’ and primer‐reverse: 5’GCCTGCCCTGATGTCTACTC3’ used for detection of genotype. Mice were housed at constant levels of temperature and humidity with a 12 h light/dark cycle and allowed free access to food and water. All experimental protocols were approved by the Experimental Animal Welfare and Ethics Review Committee of the Hebei University (Approval Number: HBULS2025002).

### 4.3 Cell Culture and Treatment

HaCaT and HEK293T cells were cultured in Dulbecco's modified Eagle's medium (DMEM) containing 10% (vol/vol) FBS, 1% streptomycin‐penicillin in a humidified incubator at 37°C and 5% CO_2_. For experiments, HaCaT cells were seeded for 12 h and then starved in DMEM for an additional 12 h, followed by stimulation with C4 cytokine cocktail, including IL‐17A (Solarbio, P00108, 10 ng/mL), OSM (Solarbio, P00067, 10 ng/mL), IL‐22 (Solarbio, P02137, 10 ng/mL), TNF‐α (Solarbio, P00029, 10 ng/mL) or combination with shikonin (MedChemExpress, HY‐N0822, 2 µM) for 24 h. Compared with the most widely accepted and well‐established in vitro model for inducing a psoriasis‐like model in keratinocytes [48], the C4 cytokine cocktail lacks IL‐1α. However, previous studies have shown that TNF‐α can largely compensate for IL‐1α function in keratinocyte inflammatory responses [49, 50], and simplified cytokine stimulation models have been successfully employed to model psoriasis‐like inflammation [51, 52].

### 4.4 Stable Cell Line Construction

HEK293T cells were transfected with PHAGE‐Flag‐TOLLIP or PHAGE‐Flag‐TOLLIP (179‐274 aa) or the PHAGE‐Flag empty vector along with the packaging vectors pSPAX2 and pMD2.G. The shRNA lentiviruses were packaged by co‐transfection of the PLKO.1 or PLKO.1‐shTOLLIP vector together with pSPAX2 and pMD2.G into HEK293T cells. After transfection for 6 h, the medium was changed to fresh medium (10% FBS, 1% streptomycin‐penicillin). After 48 h, supernatants were collected to infect HaCaT cells. Infected cells were selected with 1 µg/mL puromycin (A1113803; Gibco) to obtain the stable cell lines.

### 4.5 Isolation and Culture of Primary Mouse Keratinocytes

Primary mouse keratinocytes were isolated from neonatal mouse skin. Briefly, peeled skin tissues were washed with PBS and then incubated with mouse epidermal keratinocyte complete culture medium (FuHeng, PY‐M076) containing 1.2 U/mL dispase II (Solarbio, D6430) overnight at 4°C. The next day, the epidermal and dermal layers were separated via sterile forceps. The epidermis was cut into small pieces and further digested in 0.1% collagenase I (Solarbio, C8140) at 37°C for 1 h. The tissue suspension was filtered through a 100‐µm cell strainer. The isolated cells were washed twice with PBS and resuspended in the mouse epidermal keratinocyte complete culture medium before seeding into dishes for subsequent experiments.

### 4.6 EdU Assay

Cell proliferation was assessed using an EdU incorporation assay according to the manufacturer's instructions (RIBOBIO, C10310‐3). Briefly, cells were incubated with EdU staining buffer for 2 h, then fixed with 4% paraformaldehyde. The cells were subsequently analyzed by flow cytometry using a CytoFLEX instrument (Beckman Coulter, USA).

### 4.7 Cell Viability Assay

Cell Counting Kit‐8 (CCK‐8) (APExBIO, K1018) was used to measure cell viability following the manufacturer's guidelines. Cells (20 000 per well) were seeded in 96‐well plates and allowed to adhere for 12 h prior to treatment. 1:10 dilution of CCK‐8 reagent was added to each well and incubated at 37°C with 5% CO_2_ for 1 h. Absorbance values at 450 nm were measured using Multiskan Go (Thermo Fisher Scientific, USA).

### 4.8 Detection of Glucose Consumption, Lactate Production

Cells were seeded in 60 mm plates, incubated for 12 h, and then starved for 12 h. Subsequently, the cells were stimulated with C4 alone or in combination with shikonin for 24 h, and then the supernatant of the culture medium was collected for measurement of glucose and lactate concentrations. The levels of glucose were determined using the Glucose Kit (Nanjing Jiancheng Bioengineering Institute, A154‐1‐1) and the levels of lactate were determined using the Lactate Assay Kit (Nanjing Jiancheng Bioengineering Institute, A019‐2‐1) according to their respective manufacturer's protocols. At the same time, the number of cells in each plate was counted. Glucose consumption and lactate production were normalized to cell number, respectively.

### 4.9 Pyruvate Kinase Activity Assay

Pyruvate kinase activity was determined using the Pyruvate Kinase Activity Assay Kit (Solarbio, BC0545) according to the manufacturer's instructions. Briefly, cells from 60‐mm dish were collected, extracted with 1 mL of assay buffer, and then centrifuged to yield a clarified extract. 10 µL of extract, 10 µL of substrate, and 180 µL of reaction mixture were added per well, and the absorbance at 340 nm was measured at baseline (A1) and following incubation for 1 min at 37°C (A2). PK activity was calculated based on the provided instructions.

### 4.10 Extracellular Acidification Rate Assay

Extracellular acidification rate (ECAR) measurements were performed using the XF24 Extracellular Flux Analyzer (Agilent, USA) with Glycolysis Stress Test kit (Agilent, no.103020‐100). The day before this experiment, cells were seeded at 20 000 per well in XF24 cell culture microplates. The next day, following the indicated stimulation, the medium was replaced with XF DMEM Medium (pH 7.4). Cells were then incubated in a CO_2_–free incubator at 37°C for 1 h to equilibrate pH and temperature. Changes in extracellular acidification rate were measured following treatment with glucose (10 mM), oligomycin (1 µM), and 2‐deoxyglucose (50 mM). The ECAR values were normalized based on cell numbers. Data were processed and analyzed using the Seahorse Wave software.

### 4.11 Transcriptome Data Acquisition and Analysis

mRNA sequencing data and corresponding clinical information for human psoriasis and non‐psoriasis samples were obtained from the Gene Expression Omnibus (GEO) database (https://www.ncbi.nlm.nih.gov/gds). Differential expression analysis and gene‐gene correlation studies were performed on lesional skin, non‐lesional skin from psoriatic patients, and healthy control skin.

### 4.12 Single‐Cell RNA Sequencing Analysis

Single‐cell transcriptomic analysis was performed on the publicly available dataset GSE228421 downloaded from the GEO database. All bioinformatic analyses were conducted using R software (version 4.3.1) with the Seurat package (v5.0.1) for raw data reading, quality control, normalization, dimensionality reduction, cell clustering, and visualization.

Briefly, raw expression matrices in 10× Genomics format were imported, and Seurat objects were constructed. Samples were assigned to the control group and experiment group according to the provided sample annotation. Cells were filtered for quality control; eligible cells were retained with the number of detected genes ranging from 200 to 5000 and the mitochondrial gene proportion was lower than 20%. After filtering, data were normalized, highly variable features were identified, and scaling was performed followed by principal component analysis (PCA). The Harmony algorithm was applied to eliminate batch effects among multiple samples. Uniform Manifold Approximation and Projection (UMAP) was used for dimensionality reduction and visualization based on batch‐corrected results, and the Louvain algorithm was adopted for cell clustering. Cell type annotation was implemented via the SingleR package with the Human Primary Cell Atlas as the reference dataset. The DimPlot function was used to generate UMAP plots to display cell distribution in the control and experiment groups, with cells colored according to annotated cell types. The FeaturePlot function was utilized to visualize the spatial expression pattern of the target gene *TOLLIP* across the two groups. The DotPlot function was used to construct dot plots to illustrate the average expression level and cell expression proportion of *TOLLIP* together with psoriasis‐related marker genes (*KRT5*, *KRT6A*, *KRT14*, *KRT16*, *S100A8*, *S100A9*). All UMAP plots, feature distribution plots, and dot plots were visualized using the ggplot2 package (v3.4.4) and patchwork package (v1.1.2).

### 4.13 Imiquimod‐Induced Psoriatic Mouse Model

The dorsal skin of the female mice (7–9 weeks of age) from the same litters and cages was depilated using surgical scissors and hair removal cream. The depilated mouse dorsal skin was treated daily for 5 consecutive days with topical application of 62.5 mg commercially available 5% IMQ cream (Sichuan Med‐Shine Pharmaceutical Co., LTD, China). As a control for IMQ, the same volume of Vaseline (Haishi Hainuo, China) was also applied to the skin. On day 6, the back skin was isolated for respective experimental analysis.

The severity of psoriasis was monitored and graded using the clinical PASI score for psoriasis patients. Erythema, scaling, and thickness were scored independently on a scale of 0–4: 0, none; 1, slight; 2, moderate; 3, marked; and 4, very marked. The scoring was performed in a fully blinded fashion. The cumulative score was obtained by adding the 3 index scores (0–12) [25, 44].

### 4.14 Adeno‐Associated Viruses (AAV) Generation and Delivery

Constructs containing the K14 promoter driving expression of mouse *Tollip* or *Tollip* (179‐274 aa) were generated by Vigene Biosciences (Shandong, China). These constructs were packaged into AAV9 virions to generate the corresponding AAV9 particles. For negative control, an empty AAV9 vector (without the transgene) was prepared using the same strategy. 3‐week‐old mice received a subcutaneous injection of AAV9 at a total dose of 3 × 10^11^ viral genomes (vg) per mouse for dorsal skin, or 1 × 10^11^ vg per mouse for auricular skin. After 5 weeks of AAV injection, mice were treated daily for 5 consecutive days with 62.5 mg (for dorsal skin) or 25 mg (for auricular skin) of commercially available 5% IMQ cream to the shaved skin to evaluate the effects.

### 4.15 Hematoxylin and Eosin (H&E) Staining

Skin tissues were fixed in 4% paraformaldehyde, embedded in paraffin, and sectioned at 5 µm thickness. For H&E staining, sections were deparaffinized, stained with hematoxylin for 5 min, followed by eosin for 30 s.

### 4.16 Immunohistochemistry (IHC)

Paraffin‑embedded skin sections were deparaffinized in 100% (vol/vol) xylene and rehydrated in descending concentrations of ethanol. Antigen retrieval was performed in Tris‐EDTA buffer (pH 9.0) using a microwave oven at 95°C for 15 min. Sections were then incubated in blocking buffer containing 8% goat serum for 1 h, followed by overnight incubation at 4°C with primary antibodies: Ki67 (1:1000; Proteintech, 27309‐1‐AP), F4/80 (1:5000; Proteintech, 29414‐1‐AP), CD3 (1:3500; Proteintech, 17617‐1‐AP) and TOLLIP (1:500; Proteintech, 11315‐1‐AP). After washing, sections were incubated with HRP‐conjugated goat anti‐rabbit IgG (1:1000; Servicebio, GB23303) for 30 min at room‐temperature, followed by diaminobenzidine (DAB) substrate for 5 min. Images were captured using a light microscope (Primostar 3, ZEISS).

### 4.17 Immunofluorescence (IF)

Sections were deparaffinized, rehydrated, and subjected to antigen retrieval as described for IHC. After blocking with 8% goat serum for 1 h, sections were incubated overnight at 4°C with Alexa Fluor 488‐conjugated anti‐Flag antibody (1:1000; Aladdin, Ab218449) and rabbit anti‐Keratin 5 antibody (1:500; Proteintech, 26411‐1‐AP). The following day, sections were incubated with Dylight 594‐conjugated goat anti‐rabbit IgG (1:1000; Abbkine, A23420) for 1 h at room‐temperature. Nuclei were stained with DAPI (Solarbio, ID22502). Images were captured using an inverted biological microscope (IX73, OLYMPUS).

### 4.18 Western Blot and Antibodies

Cell and skin samples were collected and lysed using 1×SDS lysis buffer (650 mM Tris [pH 6.8], 2% SDS, 20% glycerol) and NETN 300 buffer (20 mM Tris–HCl [pH 7.4], 300 mM NaCl, 1 mM EDTA, 0.5% NP‐40), respectively. After lysis, the samples were centrifuged at 12 000 rpm for 10 min at 4°C. The supernatant was collected, and protein concentration was determined using a bicinchoninic acid (BCA) assay kit (NCM biotech, WB6501). Subsequently, the supernatants were mixed with SDS loading buffer (10% SDS, 5% β‐mercaptoethanol, 50% glycerol, 0.5% bromophenol blue, 0.25 M Tris–HCl [pH 6.8]) and boiled at 95°C for 10 min. Proteins were separated by SDS‐PAGE and transferred to PVDF membranes. The membranes were blocked with 5% non‐fat milk and then incubated with the indicated primary antibodies. Signal detection was performed using a chemiluminescent substrate and visualized with a ChemiDoc MP Imaging System (Bio‐Rad). β‐Actin and α‐Tubulin were used as internal controls for cell samples and mouse skin samples, respectively. Densitometric analysis was performed using ImageJ software.

Antibodies used in this study are listed as follows: goat anti‐rabbit IgG (H+L) (1:5000; ZB‐2301), goat anti‐mouse IgG (H+L) (1:5000; ZB‐2305), and β‐actin (1:1000; TA‐09) were purchased from ZSGB‐BIO. p‐ERK1/2 (1:2000; 4370), p‐JNK (1:1000; 4668) and p‐p38 (1:1000; 4511) were purchased from Cell Signaling Technology. PKM2 (1:10000; 60268‐1‐Ig), TOLLIP (1:5000; 11315‐1‐AP), JNK (1:10000; 66210‐1‐Ig), p38 (1:5000; 14064‐1‐AP), ERK1/2 (1:5000; 11257‐1‐AP), α‐Tubulin (1:5000; 11224‐1‐AP), HA (1:10000; 66006‐2‐Ig), Myc (1:5000; 60003‐2‐Ig) were purchased from Proteintech. Flag (1:10000; M185‐3L) was purchased from MBL Beijing Biotech. p‐p65 (1:1000; 310013), p65 (1:1000; R380172) were purchased from Zenbio.

### 4.19 RNA Extraction and Quantitative Real‐Time PCR (qPCR)

RNAs were extracted with TRIzol Reagent (Tiangen) according to the manufacturer's protocol. RNA integrity and purity were assessed by agarose gel electrophoresis and spectrophotometric measurement (NanoDrop), respectively. cDNA was synthesized using the HiScript III RT SuperMix for qPCR (+gDNA wiper) kit (R323, Vazyme) following the manufacturer's instructions. qPCR was performed on a CFX96 Real‐Time System (Bio‐Rad, USA), and the relative gene expression levels were calculated using the 2^–ΔΔCt method. The primers used in this study are listed in Tables S1 and S2.

### 4.20 Pathway Enrichment Analysis

Mass spectrometry data from our previous study of protein TOLLIP were analyzed [21]. Differential expression was defined using a log_2_ fold change (log_2_FC) threshold of ≥ 1.2 when comparing A‐overexpressed samples to controls. Pathway enrichment analysis was performed using the Database for Annotation, Visualization, and Integrated Discovery (DAVID; https://davidbioinformatics.nih.gov/). Wikipathways terms with a *p*‐value < 0.05 were considered statistically significant.

### 4.21 Immunoprecipitation (IP) Assay

Cells were lysed on ice for 30 min using NETN300 buffer (20 mM Tris‐HCl [pH 7.4], 300 mM NaCl, 1 mM EDTA, 0.5% NP‐40). The lysates were centrifuged at 12 000 rpm for 10 min at 4°C. The resulting supernatants were incubated with a mixture of Protein A (101142, Invitrogen) and Protein G (101243, Invitrogen) beads, together with the indicated antibodies, for 4 h at 4°C with gentle rotation. Subsequently, the beads were washed three times with 1 mL of NETN300 buffer and three times with 1 mL of NETN100 buffer (20 mM Tris‐HCl [pH 7.4], 100 mM NaCl, 1 mM EDTA, 0.5% NP‐40). Proteins bound to the beads were eluted and subjected to SDS‐PAGE separation, followed by immunoblotting analysis using the specified antibodies.

### 4.22 Ubiquitination Assay

Following transfection with the indicated plasmids, cells were collected and lysed by resuspension in 80 µL of ice‐cold IP buffer (20 mM Tris‐HCl [pH 7.4], 150 mM NaCl, 1 mM EDTA, 0.5% NP‐40) supplemented with protease inhibitor cocktail (04693132001, Roche). Then, the immunoprecipitates were resuspended in 10 µL of 10% SDS and heated at 95°C for 10 min to disrupt non‐covalent interactions. The eluates were then diluted ten‐fold with IP buffer and further lysed by ultrasonication (Sonics). Subsequent steps were identical to the standard IP assay protocol.

### 4.23 GST‐Pull Down Assay

Recombinant GST fusion proteins (GST, GST‐TOLLIP) were expressed in *E. coli* BL21 (DE3) competent cells. Protein expression was induced, and cells were harvested by centrifugation at 4000 × g for 10 min at 4°C. The cell pellets were resuspended in lysis buffer (25 mM Tris–HCl pH 8.0, 500 mM NaCl, 5% glycerol) supplemented with 2 mM PMSF as a protease inhibitor and 2 mM β‐mercaptoethanol as a reducing agent. Cell disruption was performed by sonication on ice, followed by centrifugation at 15 000 × g for 30 min at 4°C to remove cellular debris. The supernatant was incubated with glutathione‐Sepharose beads to affinity purify the GST‐tagged proteins, as previously described [53]. After binding, the beads were washed extensively, and bound proteins were eluted using elution buffer containing 10 mM glutathione, 25 mM Tris–HCl (pH 8.0), 500 mM NaCl, 5% glycerol, 2 mM PMSF, and 2 mM β‐mercaptoethanol. All purification steps were carried out at 4°C to minimize proteolytic degradation.

For the pull‐down assay, purified GST or GST‐fusion TOLLIP proteins bound to glutathione beads were incubated with HA‐tagged PKM2, expressed in HEK293T cells, for 4 h at 4°C with gentle rotation. The beads were then washed four times with NETN100 buffer (20 mM Tris–HCl pH 8.0, 100 mM NaCl, 1 mM EDTA, 0.5% NP‐40). Subsequently, bound proteins were eluted by boiling in SDS loading buffer and analyzed by SDS‐PAGE followed by immunoblotting using anti‐GST and anti‐HA antibodies.

### 4.24 Statistical Analysis

Statistical significance was evaluated using the two‐tailed Student's *t*‐test, one‐way ANOVA, or two‐way ANOVA, followed by Bonferroni's post hoc test where appropriate. Correlation analyses were conducted using Pearson's correlation test. A p‐value of less than 0.05 was considered statistically significant. All data are expressed as mean ± standard deviation (SD). Statistical analyses and graphing were performed using GraphPad Prism version 9.5.1.

## Author Contributions

Conceived the experiments: Z.Y. and X.J. Designed the experiments: Z.Y. and C.W. Process preparation: X.J., G.M., N.S., Q.H., X.H., K.F., and Z.Q. Testing: X.J., G.M., N.S., Q.H., and X.H. Verification test: K.F., Z.Q., and Z.T. Data processing and analysis: X.J., G.M., Q.H., Y.W., and X.L. Writing – original draft: Z.Y., X.J., and C.W. Writing – review and editing: Z.Y., G.M., N.S., and C.W.

## Conflicts of Interest

The authors declare no conflicts of interest.

## Supporting information




**Supporting File**: advs76892‐sup‐0001‐SuppMat.docx.

## Data Availability

The data that support the findings of this study are available from the corresponding author upon reasonable request.
