# Supplementary material for: TOLLIP Inhibits Psoriasis Progression via Suppressing PKM2‐Mediated Glycolysis in Keratinocytes
Source: Adv Sci (Weinh). 2026 Jul 31:e23390. Online ahead of print. doi: 10.1002/advs.202523390 (PMC13426103; doi:10.1002/advs.202523390)
Supplement: Supplementary file 1 — Supporting File: advs76892‐sup‐0001‐SuppMat.docx. [file ADVS-9999-e23390-s001.docx]

Supporting information

**TOLLIP inhibits psoriasis progression via suppressing PKM2-mediated glycolysis in keratinocytes**

Xiuhuan Jiang ^1^, Guoying Miao ^2^, Na Shen ^2^, Qingxia Han ^1^, Xiaoru Han ^1^, Zichang Qiao ^1^, Kexin Feng ^1^, Zhuo Tan ^1^, Yaguang Wang ^1^, Xiuhua Liu ^1^, Chen Wu ^1, 3, *^, Zhenzhen Yan ^1, *^

^1^ Baoding Key Laboratory of Cancer & Aging, College of Life Science, Hebei University, Baoding, 071002, China.

^2^ Department of Dermatology, Affiliated Hospital of Hebei Engineering University, Handan, China.

^3^ State Key Laboratory of New Pharmaceutical Preparations and Excipients, Hebei University, Baoding, 071002, China.

^*^ Corresponding author

Chen Wu (wuchen@hbu.edu.cn)

Zhenzhen Yan (yanzhzh@hbu.edu.cn)


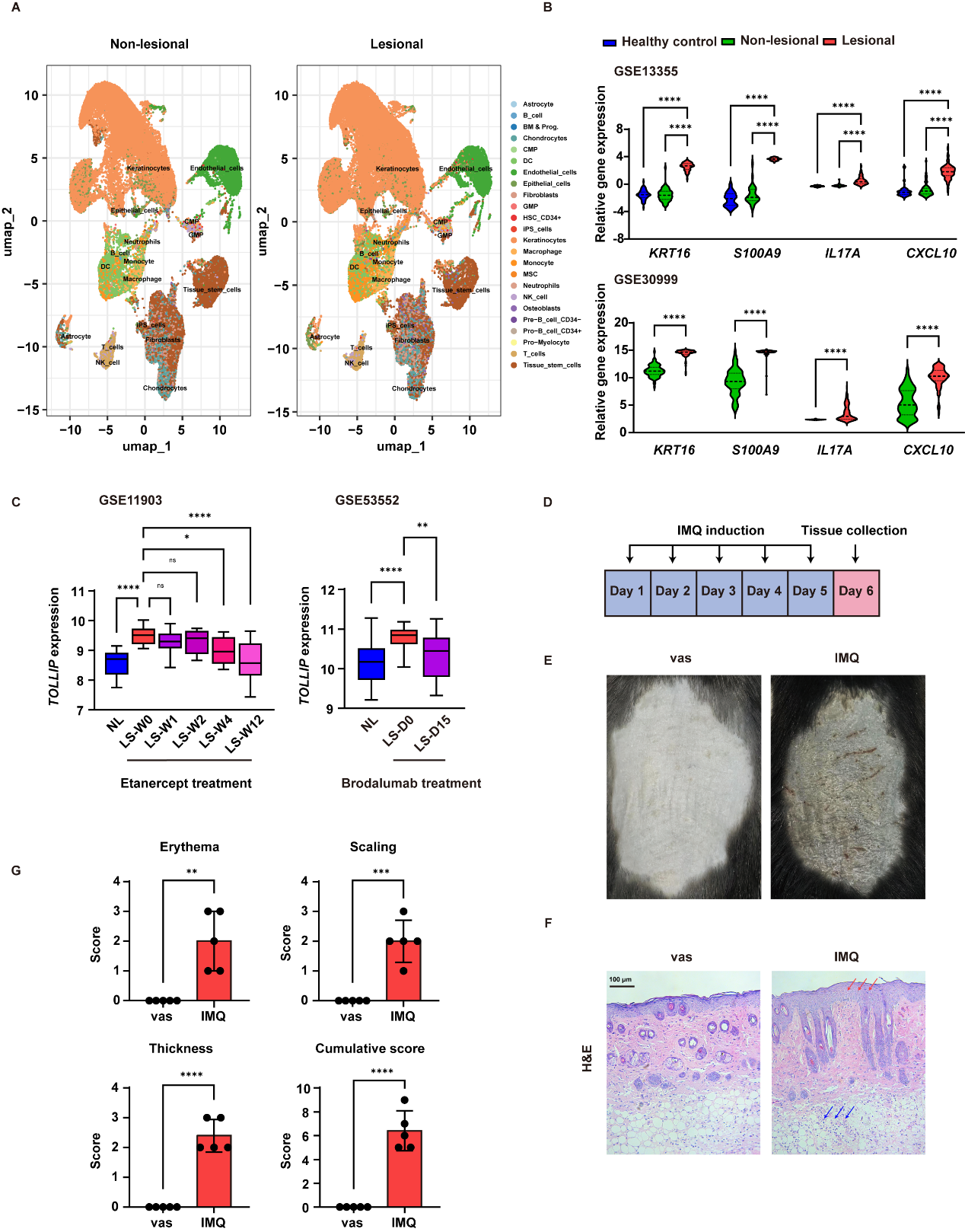


**Figure S1. TOLLIP expression is associated with psoriasis severity.**

(A) The UMAP plot shows distinct cell distribution patterns between lesional and non-lesional skin of psoriasis. Twenty-four cell types are delineated by different colors, and the cluster size reflects the cell abundance. For clarity, only the major cell types are labeled in the plot. (B) Expression levels of psoriatic markers in skin biopsy samples from healthy controls, non-lesional skin and lesional skin of psoriatic patients, as assessed through analyzing the transcriptome of published datasets: GSE13355 (healthy control, n = 64; non-lesional, n = 58; lesional, n = 58), GSE30999 (healthy control, n = 85; lesional, n = 85). (C) TOLLIP expression in non-lesional skin (NL), lesional skin (LS) and lesional skin after treatment with etanercept (TNF-α inhibitor) or brodalumab (anti-IL17RA monoclonal antibody) for indicated durations of psoriatic patients was assessed by analyzing the transcriptome of datasets: GSE11903 (NL, n = 15; LS-W0, n = 15; LS-W1, n = 15; LS-W2, n = 15; LS-W4, n = 14; LS-W12, n = 15), GSE53552 (NL, n = 24; LS-D0, n = 25; LS-D15, n = 25). W, week; D, day. (D) Schematic diagram of the IMQ-induced psoriasis mouse model study design. (E) Representative gross appearance of shaved dorsal skin of mice after 5 days of treatment with control cream or IMQ (n = 5 mice per group). (F) Representative H&E staining images of dorsal skin sections from mice subjected to control cream or IMQ (n = 3 mice per group). The red arrows represented thickened stratum spinosum and blue arrows represented inflammatory cell infiltrate. Scale bar, 100 µm. (G) Dorsal skin erythema, scaling and thickness of mice after 5 days of treatment with control cream or IMQ were scored based on the PASI scoring system (n = 5 mice per group). Data are shown as mean ± SD. ns, no significance, *p<0.05, **p<0.01, ***p<0.001, ****p<0.0001. Values were determined by two-tailed unpaired Student’s t test (B and G), one-way ANOVA followed by Bonferroni’s post hoc test (B and C).


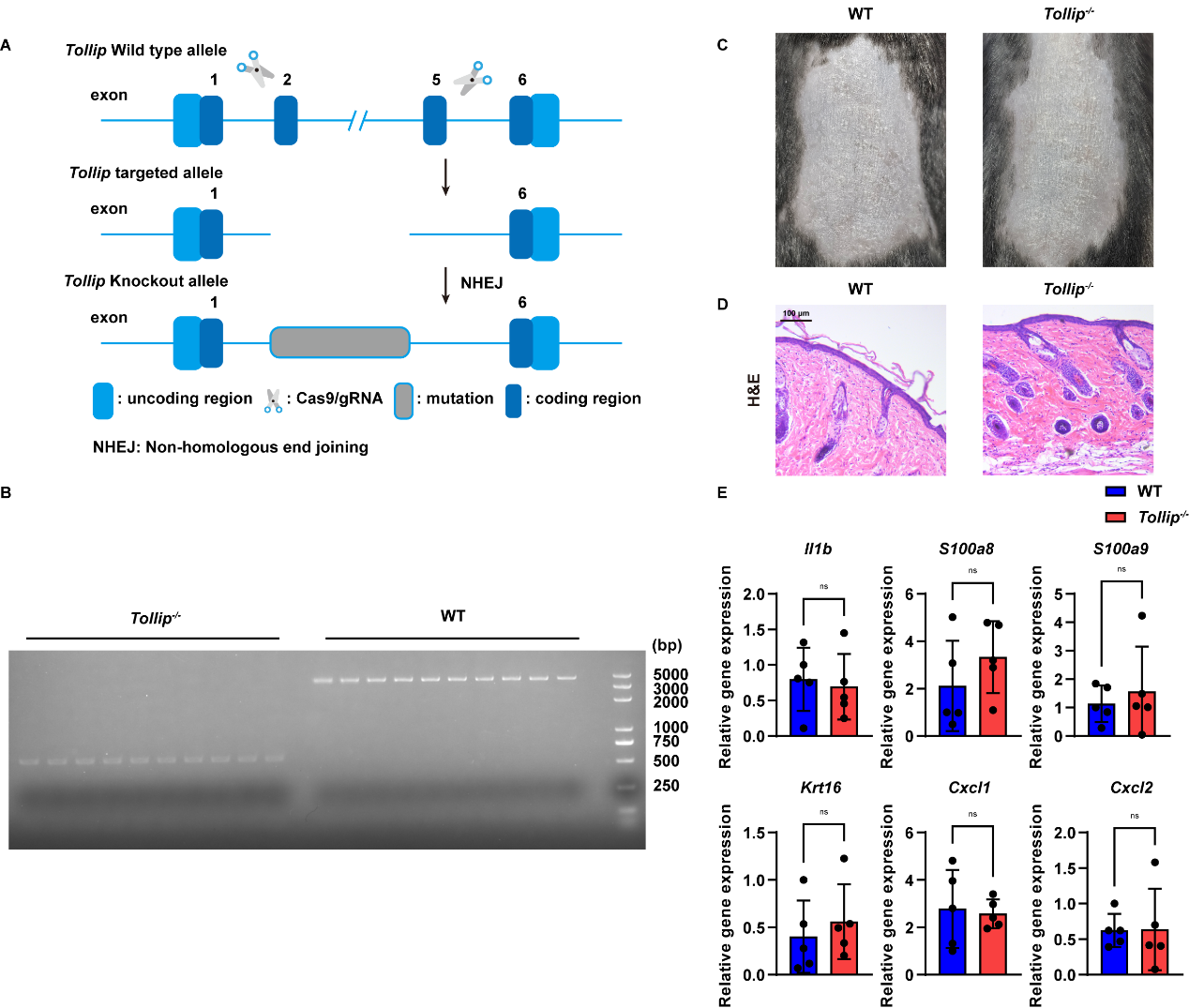


**Figure S2. Genotype identification of *Tollip*^-/-^ mice.**

(A) Construction strategy of *Tollip*^-/-^ mice. (B) PCR showing genotyping of WT and *Tollip*^-/-^ mice. (C) Representative gross appearance of shaved dorsal skin from WT and *Tollip*^-/-^ mice after 5 days of treatment with vaseline (n = 5 mice per group). (D) Representative H&E staining images of shaved dorsal skin from WT and *Tollip*^-/-^ mice after 5 days of treatment with vaseline (n = 3 mice per group). (E) qPCR analysis of *Il1b*, *S100a8*, *S100a9*, *Krt16*, *Cxcl1* and *Cxcl2* mRNA expression in skin samples from WT and *Tollip*^-/-^ mice following 5-day treatment with vaseline (n = 5 mice per group). *Tuba1a* was used as a reference gene for normalization. Data represent three independent experiments and are shown as mean ± SD. ns, no significance. Values were determined by two-tailed unpaired Student’s t test (E).


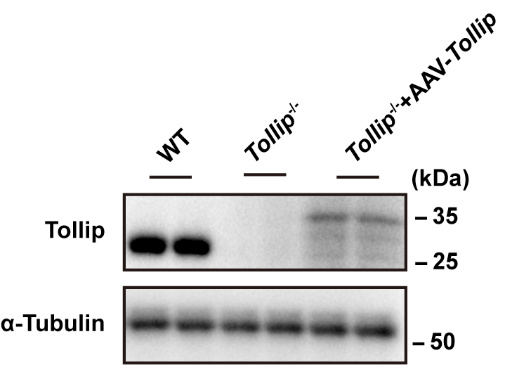


**Figure S3. Validation of TOLLIP expression in WT, *Tollip*^-/-^ and *Tollip*^-/-^+AAV-*Tollip* mice.**

Representative Western blot analysis of protein expression in skin samples from WT, *Tollip*^-/-^ and *Tollip*^-/-^+AAV-*Tollip* mice. α-Tubulin was used as a loading control. Original blots can be found in Figure S11.


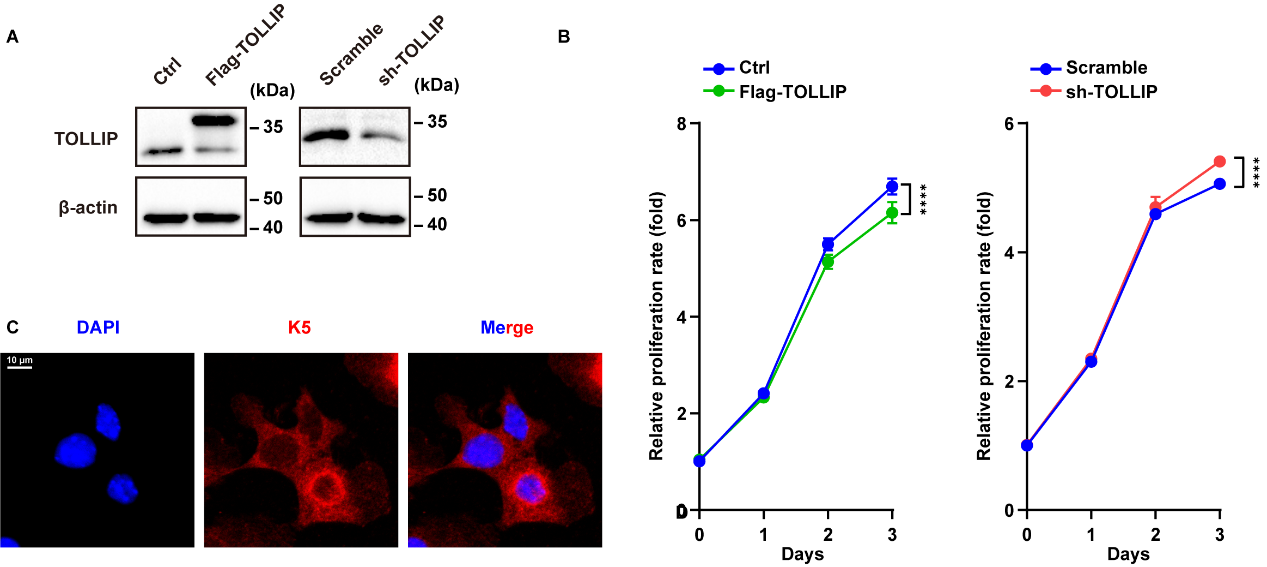


**Figure S4. TOLLIP inhibits the proliferation of keratinocytes.**

(A) Western blot analysis of TOLLIP protein levels in HaCaT cells with TOLLIP overexpression (left) or TOLLIP knockdown (right), compared to their respective control cells. β-actin was used as a loading control. Data represent three independent experiments. (B) Cell proliferation curves of HaCaT cells with TOLLIP overexpression (left) or TOLLIP-knockdown (right), along with their respective controls, following C4 treatment for 0-3 days. (C) Representative immunofluorescence images showing staining of K5 (red) and DAPI (blue) in primary mouse keratinocytes. Scale bar, 10 μm. Data represent three independent experiments and are shown as mean ± SD. ****p<0.0001. Values were determined by two-way ANOVA followed by Bonferroni’s post hoc test (B). Original blots can be found in Figure S12.


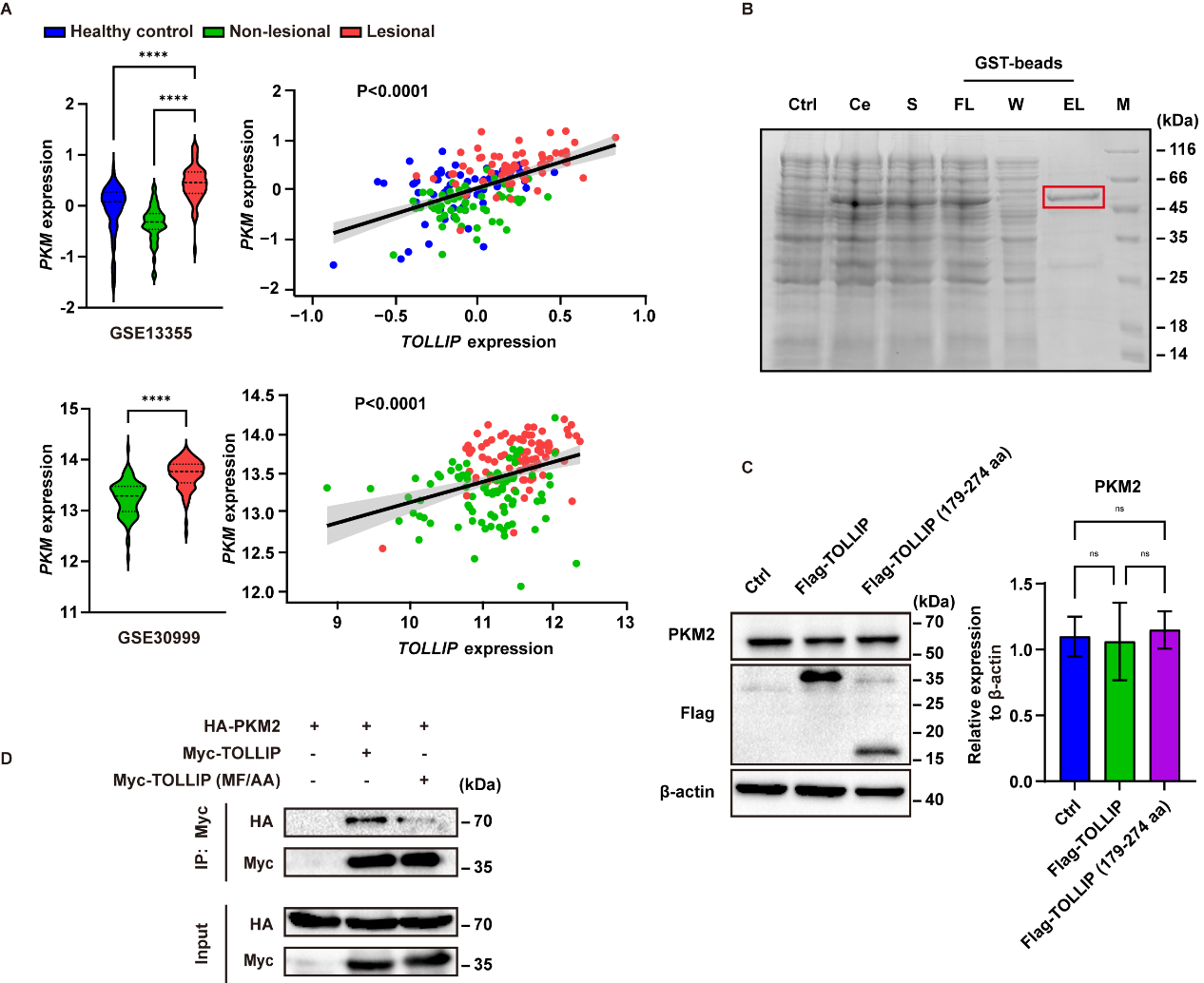


**Figure S5. TOLLIP is closely correlated with PKM2.**

(A) Analysis of public datasets (GSE13355, GSE30999) shows expression levels of PKM in healthy controls, non-lesional (NL) and lesional (LS) psoriatic skin (left), and reveals a significant Pearson correlation between TOLLIP and PKM expression across samples (right). (B) Coomassie blue staining of purified GST-TOLLIP proteins. Purified proteins from *E. coli* expression were separated by SDS-PAGE and visualized with Coomassie Brilliant Blue R-250. The red frame indicates the full-length GST-TOLLIP protein. Ctrl, whole cell lysate from uninduced bacteria (negative control); Ce, crude extract from IPTG-induced bacteria (total cell lysate); S, soluble supernatant fraction after centrifugation of the crude extract; FL, flow-through fraction (unbound proteins); W, wash fraction (non-specifically bound proteins); EL, elution fraction (purified GST-tagged proteins); M, protein molecular weight marker. (C) Western blot analysis (left) and quantification (right) of PKM2 protein expression in the control, TOLLIP-overexpressed and TOLLIP (179-274 aa)-overexpressed cells. β-actin was used as the loading control. (D) Immunoprecipitation assay showing the interaction between PKM2 and TOLLIP or TOLLIP (MF/AA) in cells transfected with the indicated plasmids. Cell lysates were immunoprecipitated with anti-Myc antibody. Data represent three independent experiments and are shown as mean ± SD. ns, no significance, ****p<0.0001. Values were determined by two-tailed unpaired Student’s t test (A), one-way ANOVA followed by Bonferroni’s post hoc test (A and C). Original blots can be found in Figure S13.


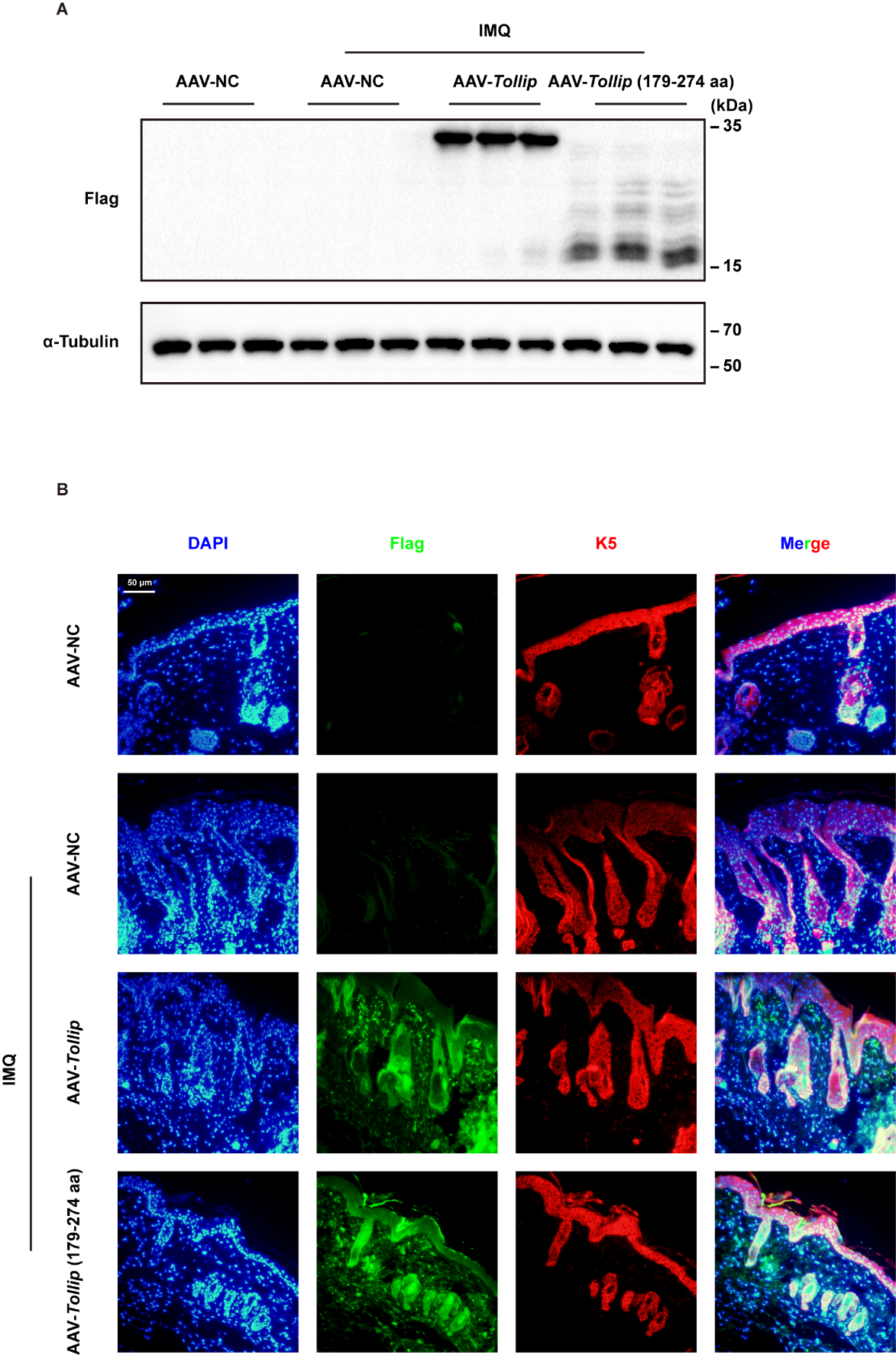


**Figure S6.** **Validation of Tollip and Tollip (179-274 aa) in skin samples from NC, Tollip- and Tollip (179-274 aa)- overexpressed mice.**

(A) Representative Western blot analysis of protein expression in skin samples from NC, Tollip- and Tollip (179-274 aa)-overexpressed mice after 5 days of treatment with control cream or IMQ. α-Tubulin was used as a loading control (n = 5 mice per group). (B) Representative immunofluorescence staining images showing Flag-positive (green) and K5-positive (red) cells in dorsal skin sections from NC, Tollip- and Tollip (179-274 aa)-overexpressed mice after 5 days of treatment with control cream or IMQ. Scale bar, 50 μm. Original blots can be found in Figure S14.


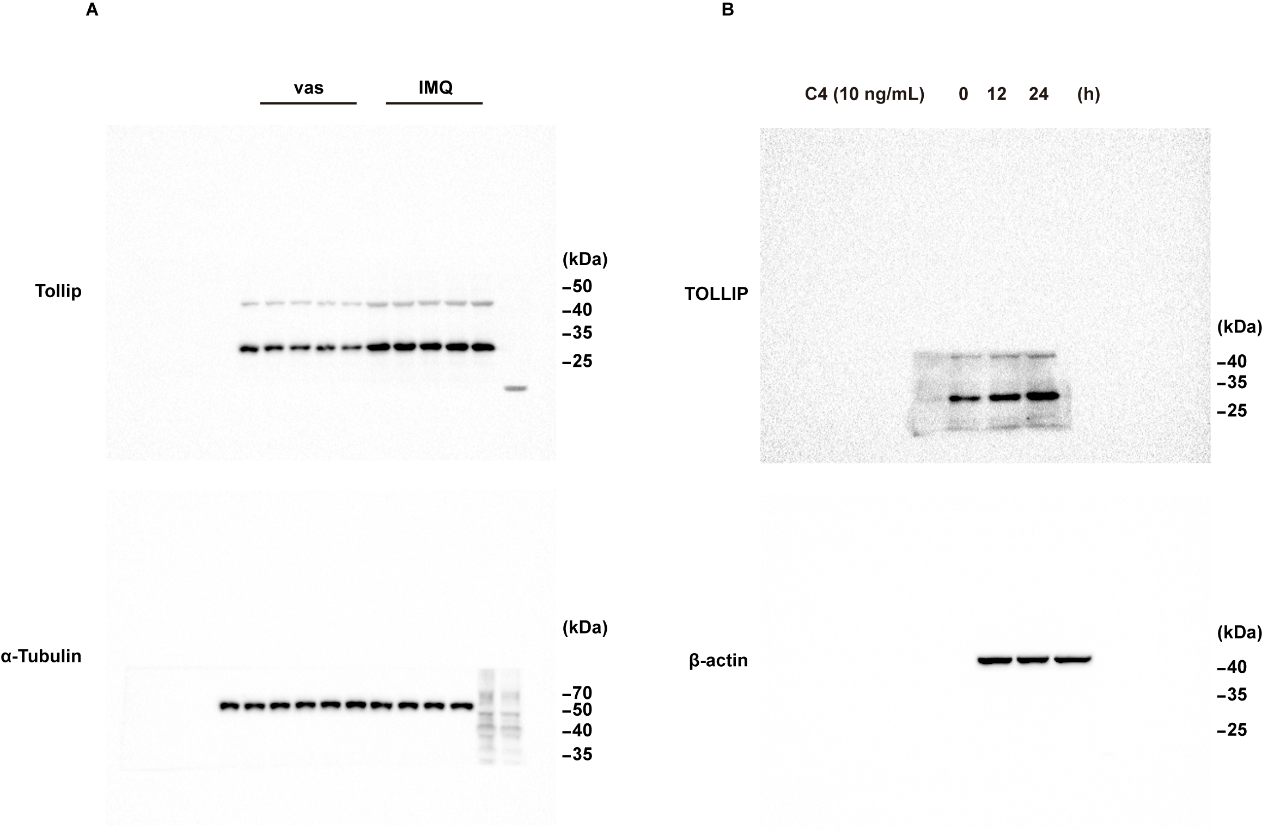


**Figure S7. Original blots for Figure 1.**

(A) Original blots for Figure 1H. (B) Original blots for Figure 1K.


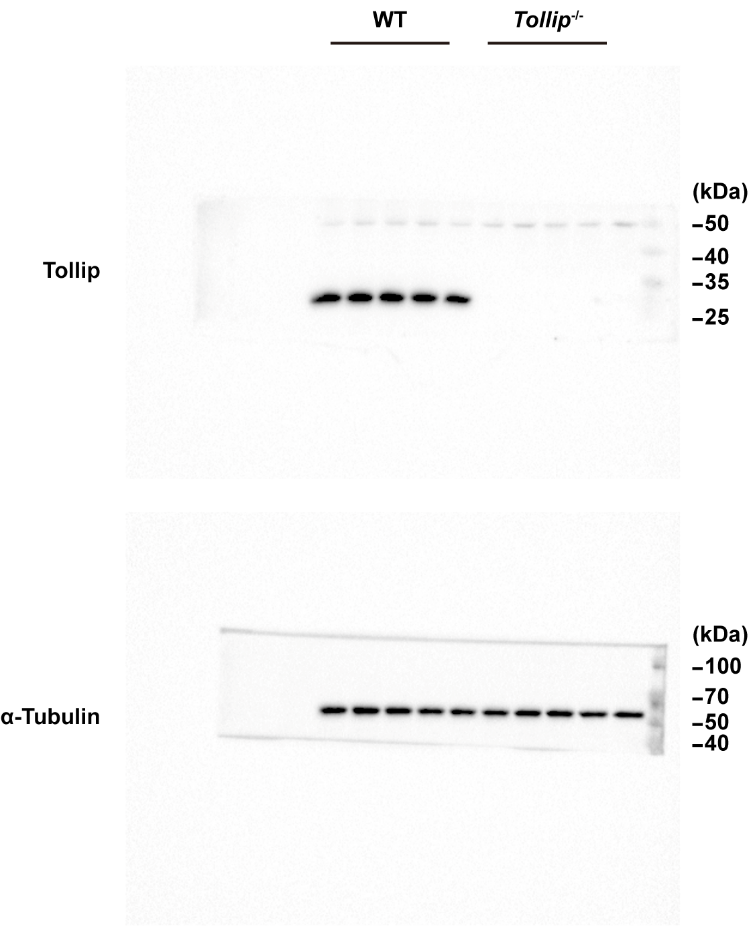


**Figure S8. Original blots for Figure 2A.**


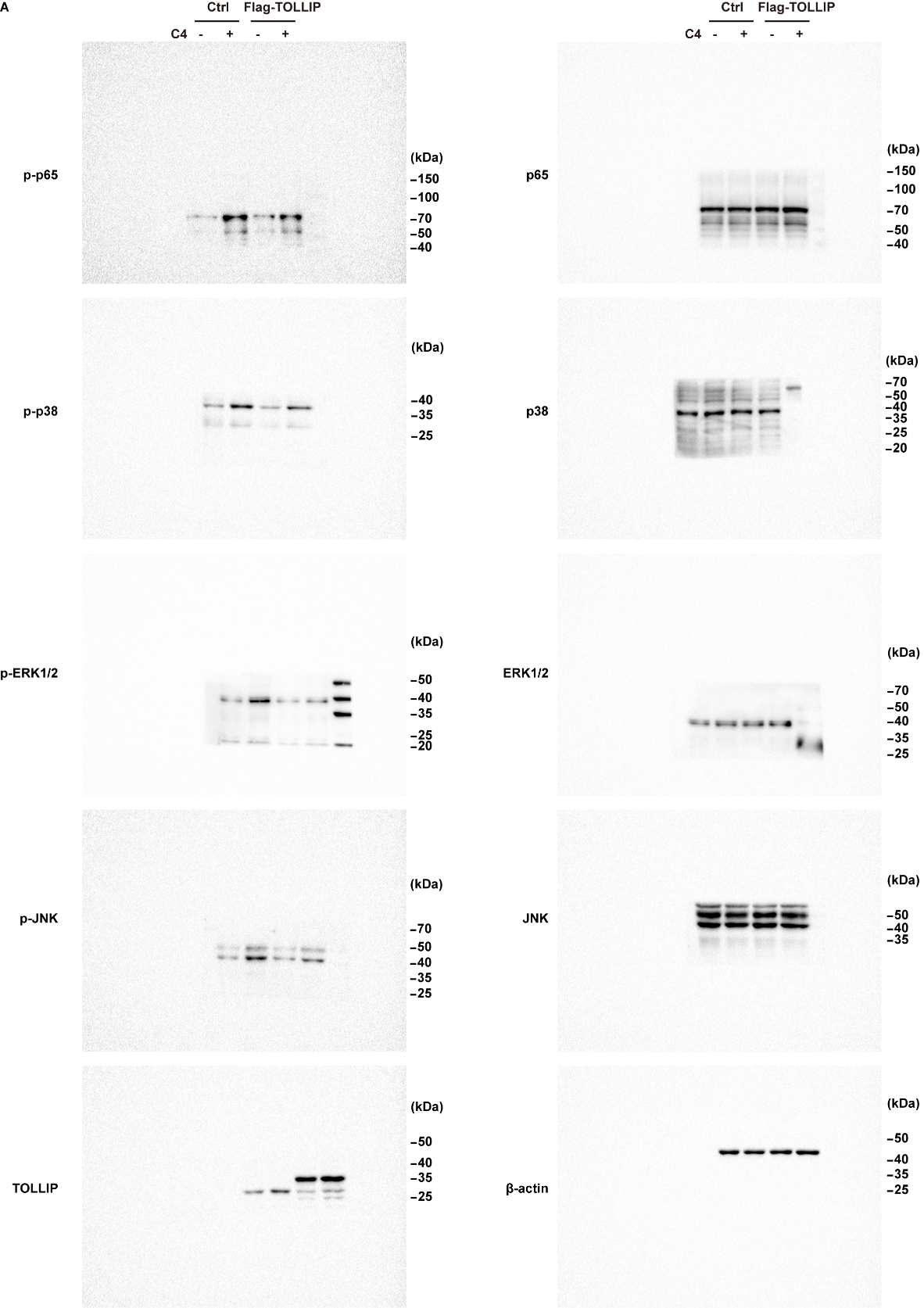


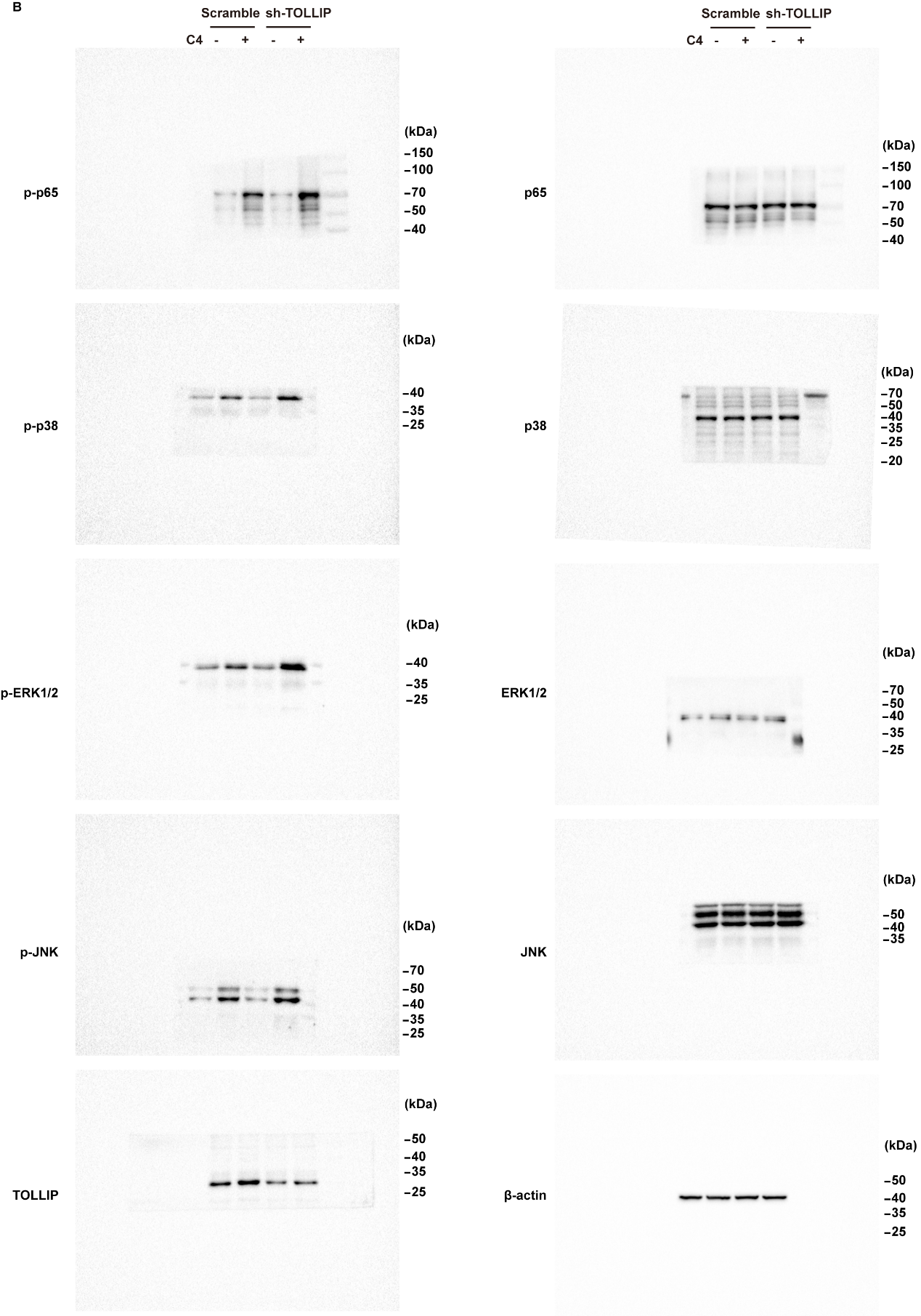


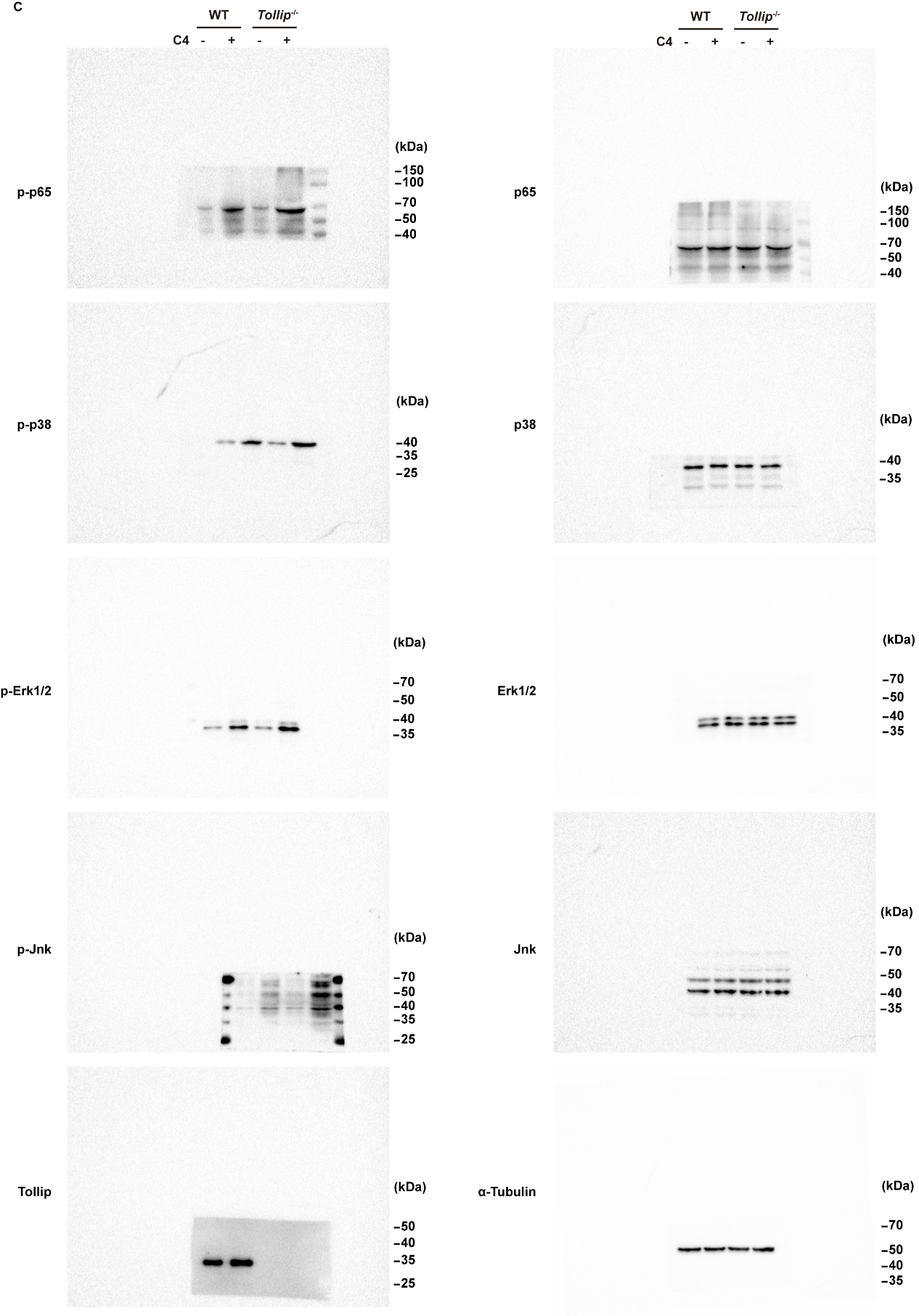


**Figure S9. Original blots for Figure 4.**

(A) Original blots for Figure 4C. (B) Original blots for Figure 4D. (C) Original blots for Figure 4G.


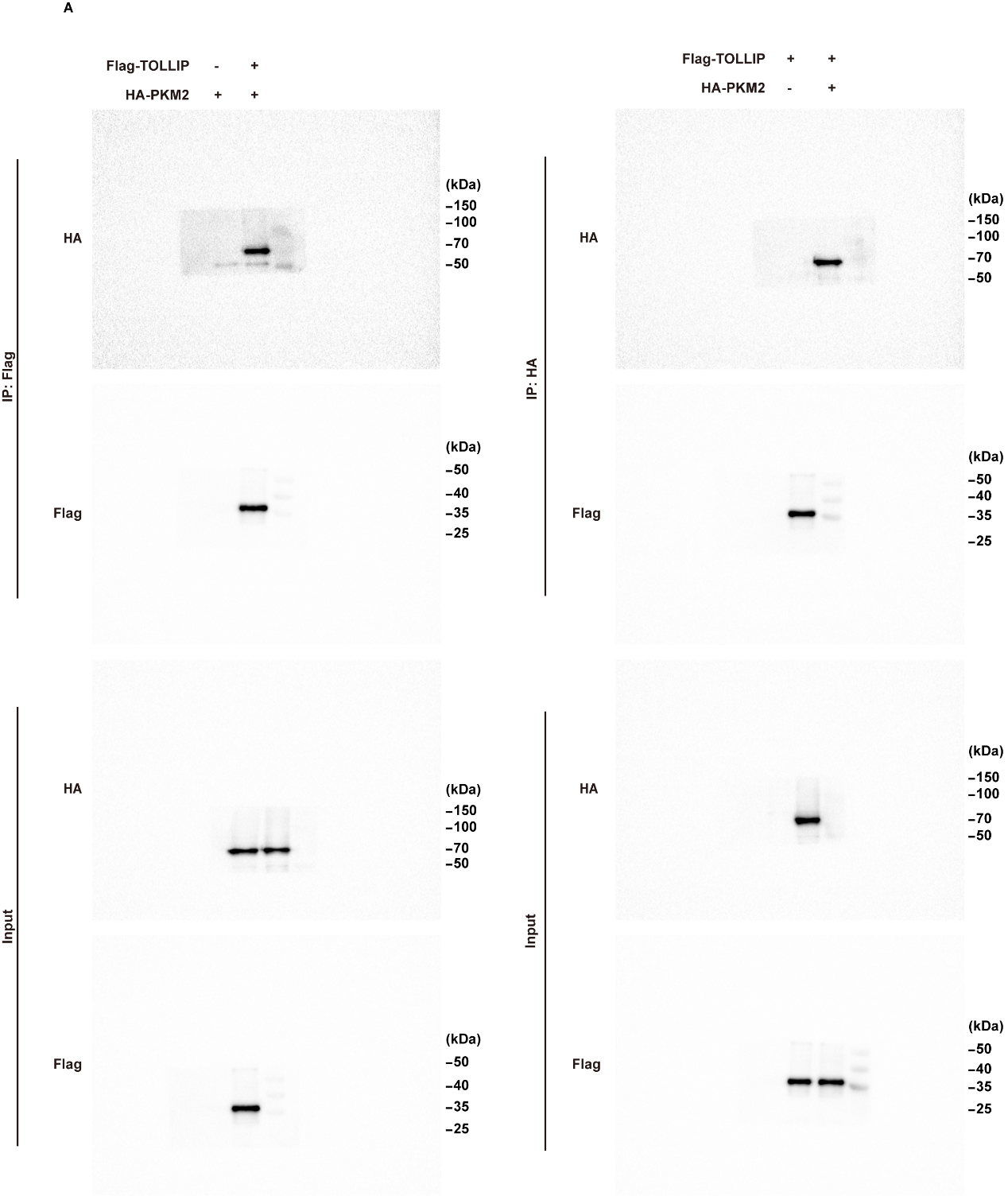


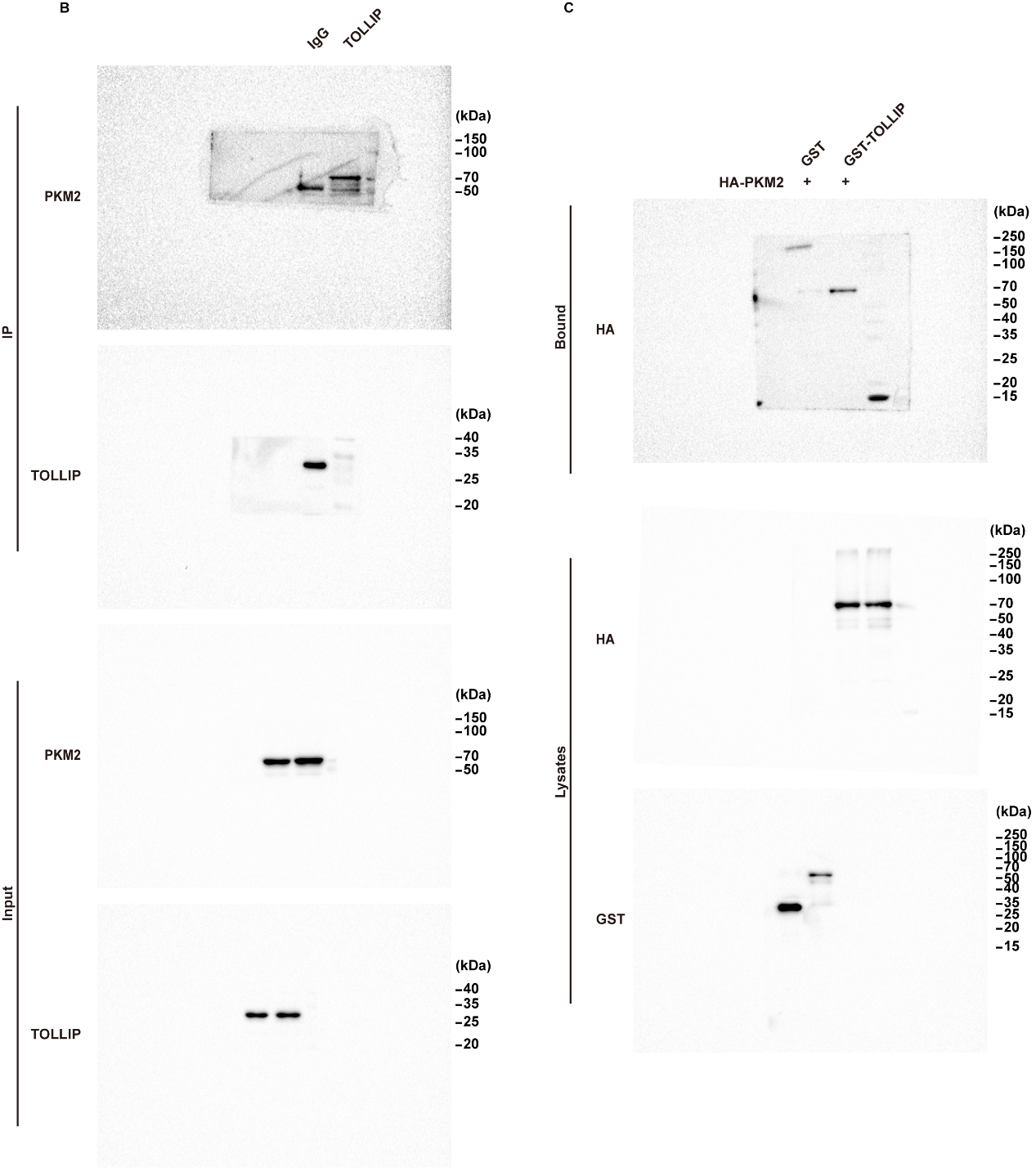


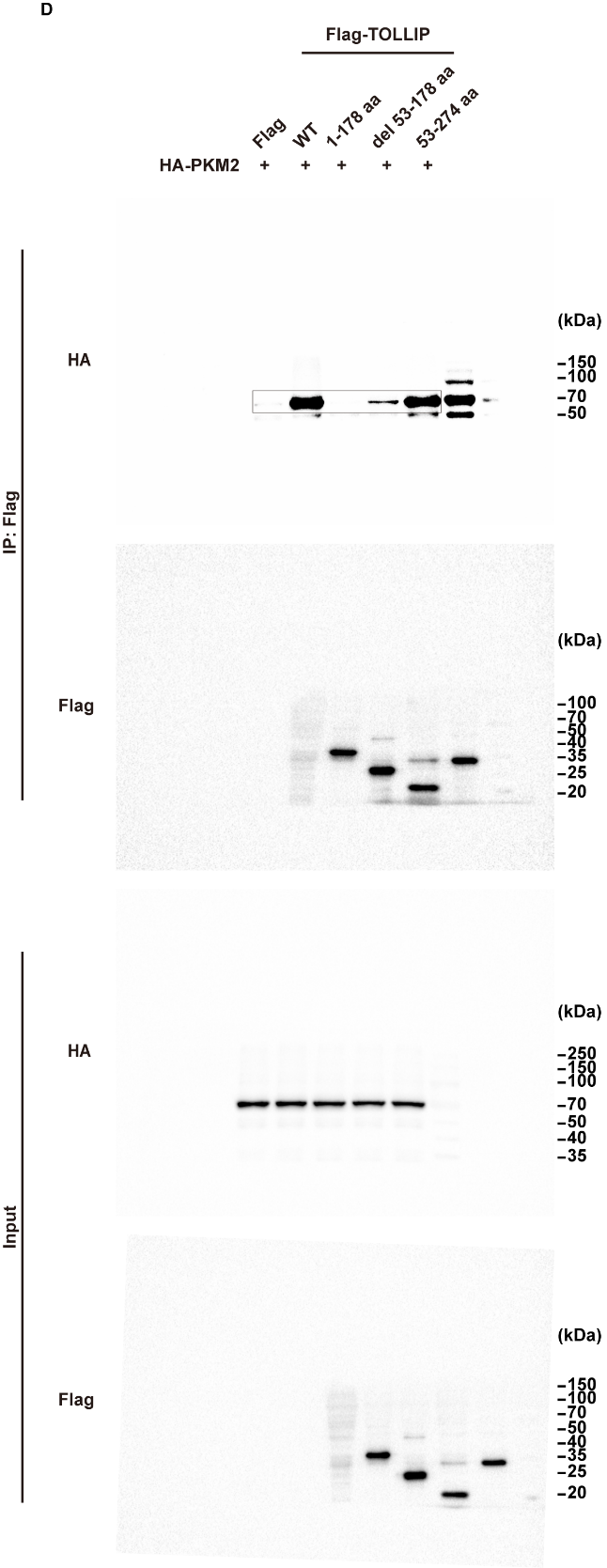


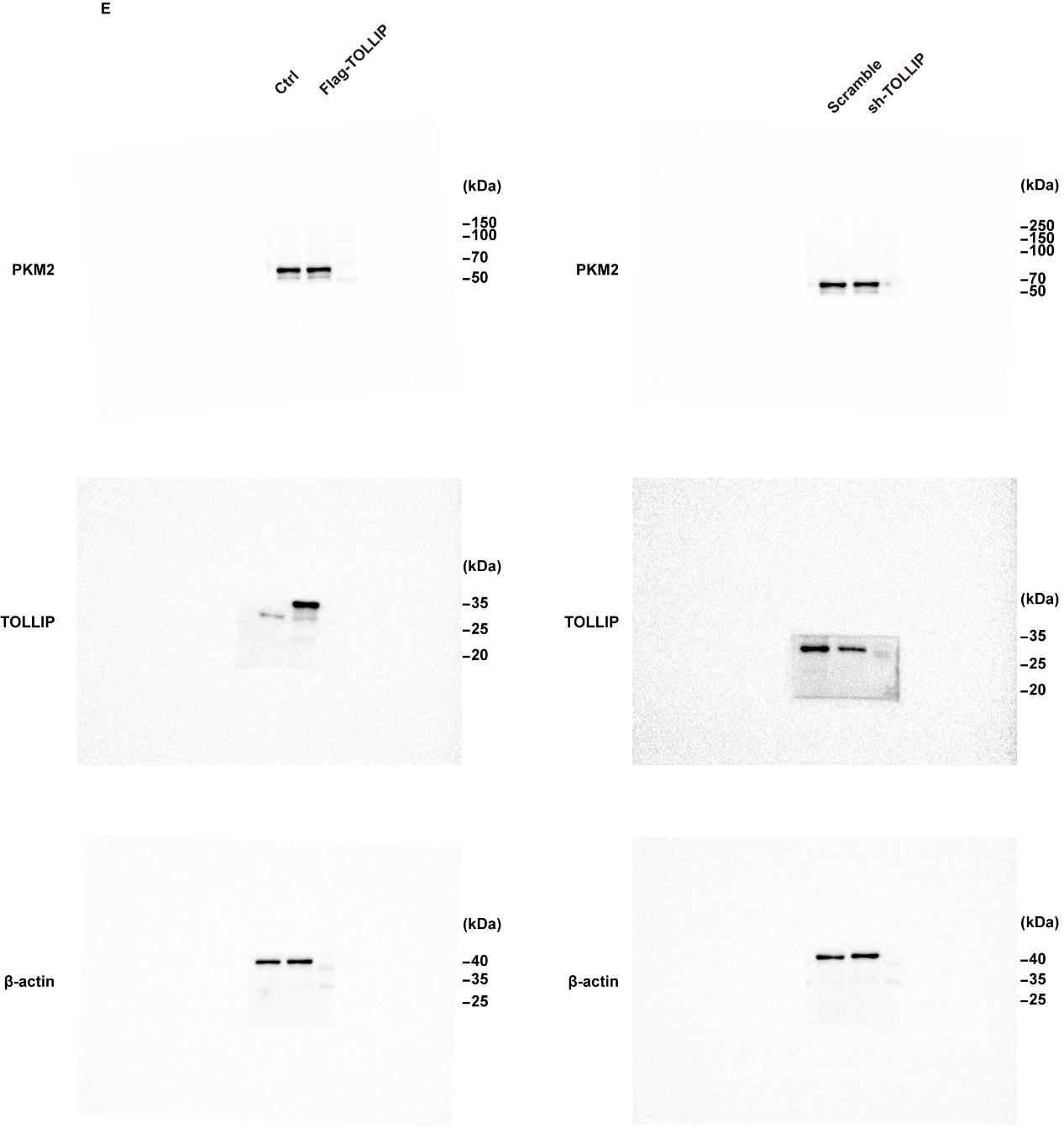


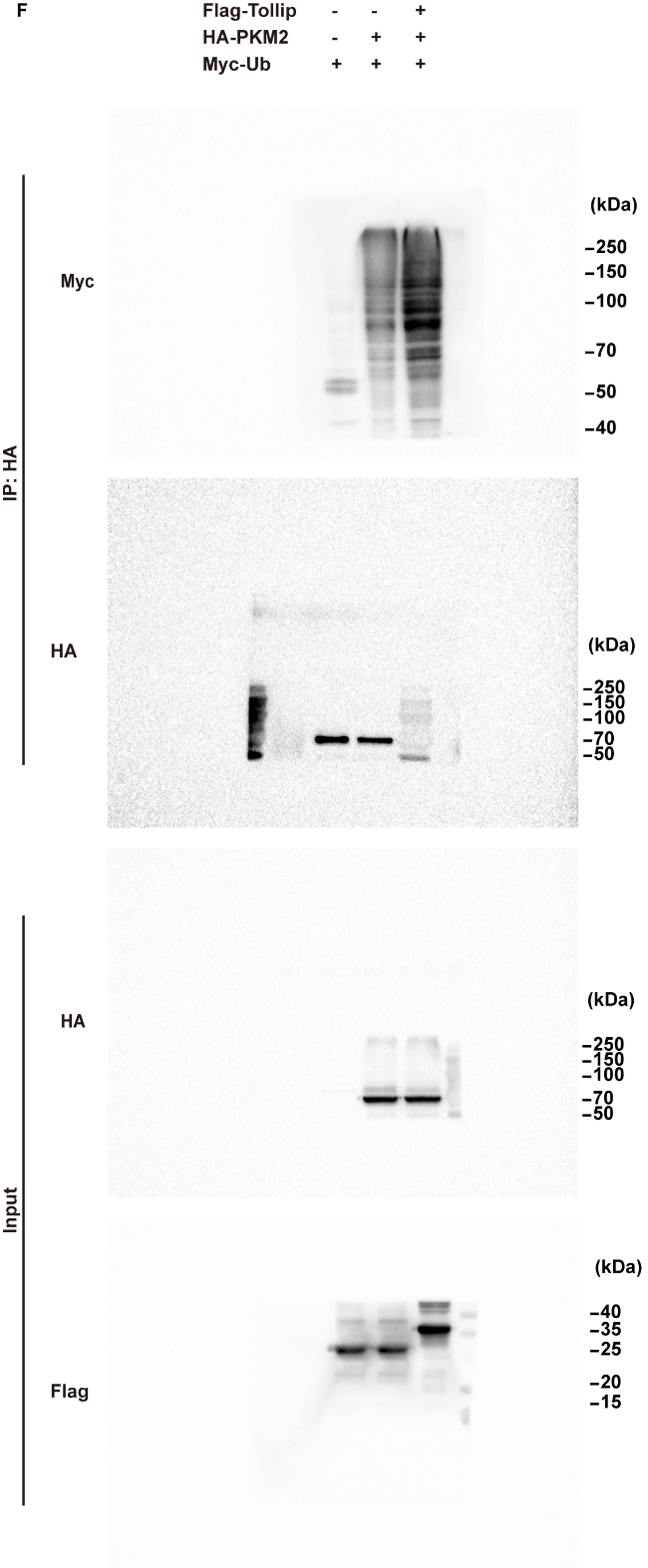


**Figure S10. Original blots for Figure 5.**

(A) Original blots for Figure 5C. (B) Original blots for Figure 5D. (C) Original blots for Figure 5E. (D) Original blots for Figure 5F. (E) Original blots for Figure 5H. (F) Original blots for Figure 5J.


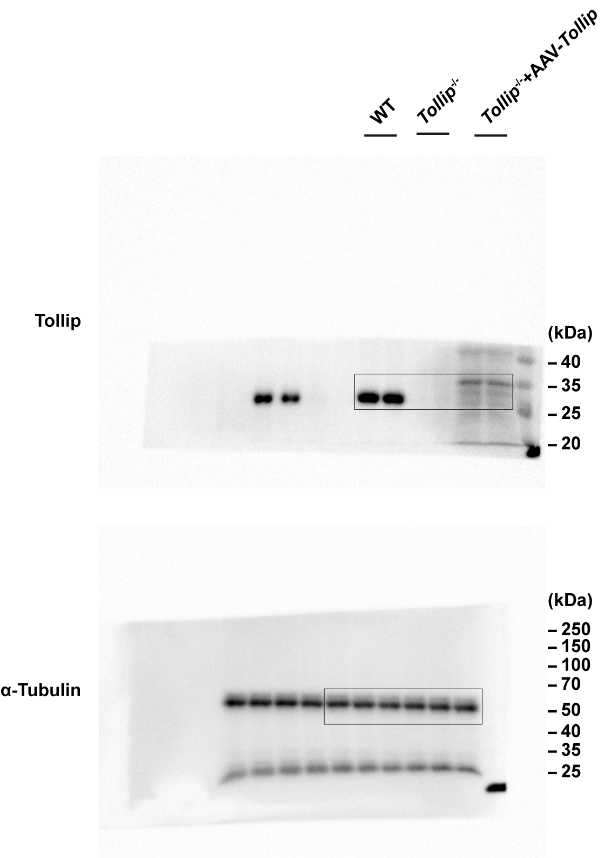


**Figure S11. Original blots for Figure S3.**


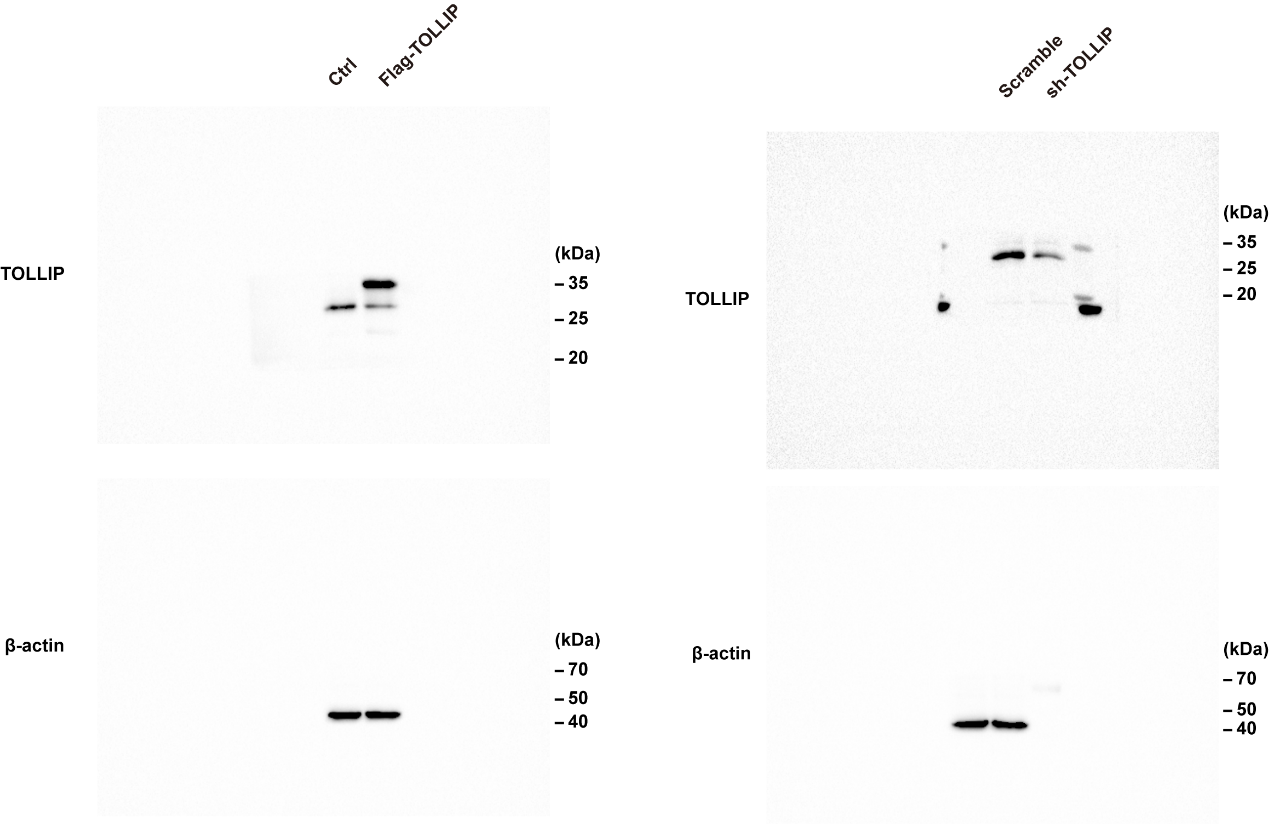


**Figure S12. Original blots for Figure S4A.**


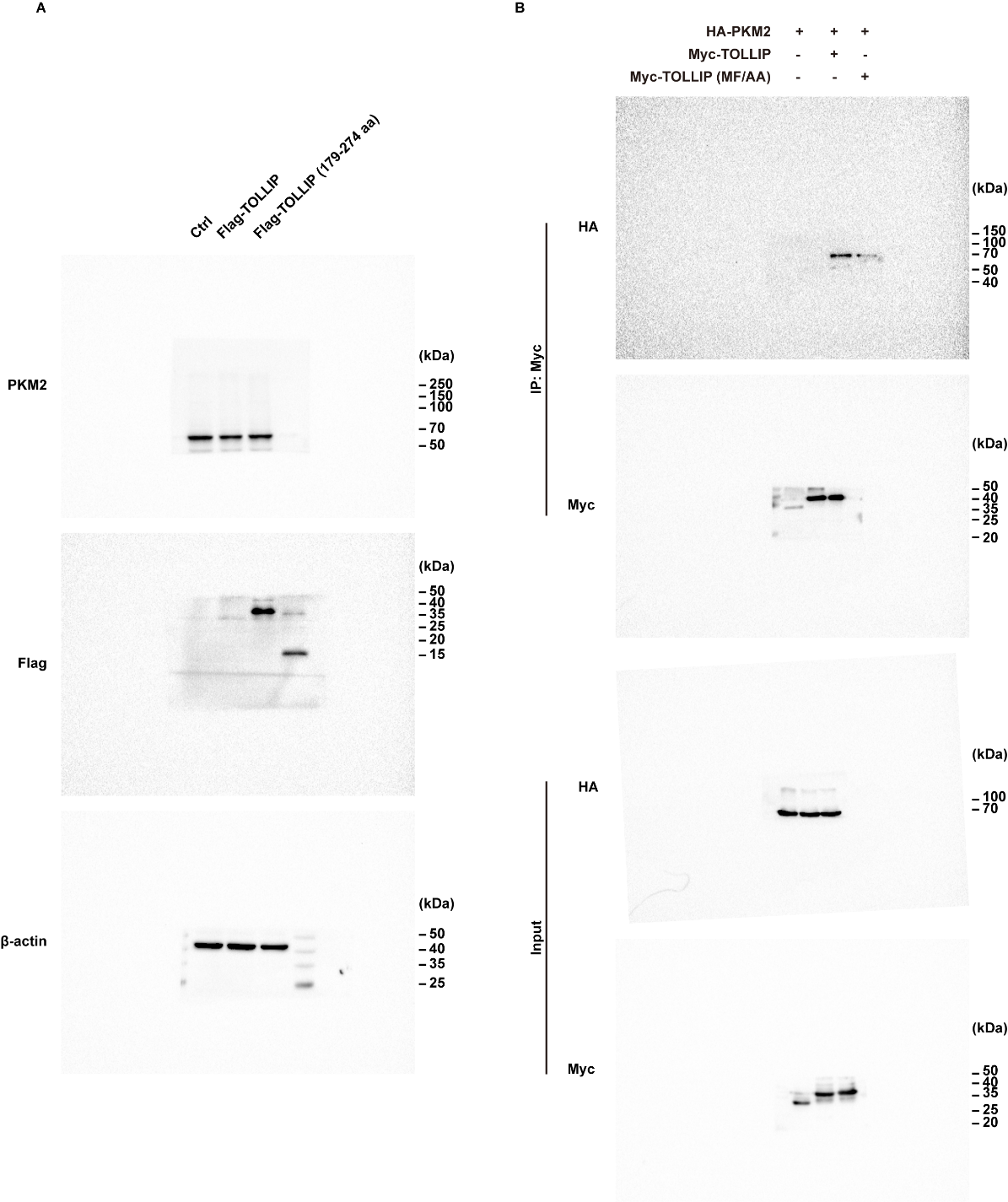


**Figure S13. Original blots for Figure S5.**

(A) Original blots for Figure S5C. (B) Original blots for Figure S5D.


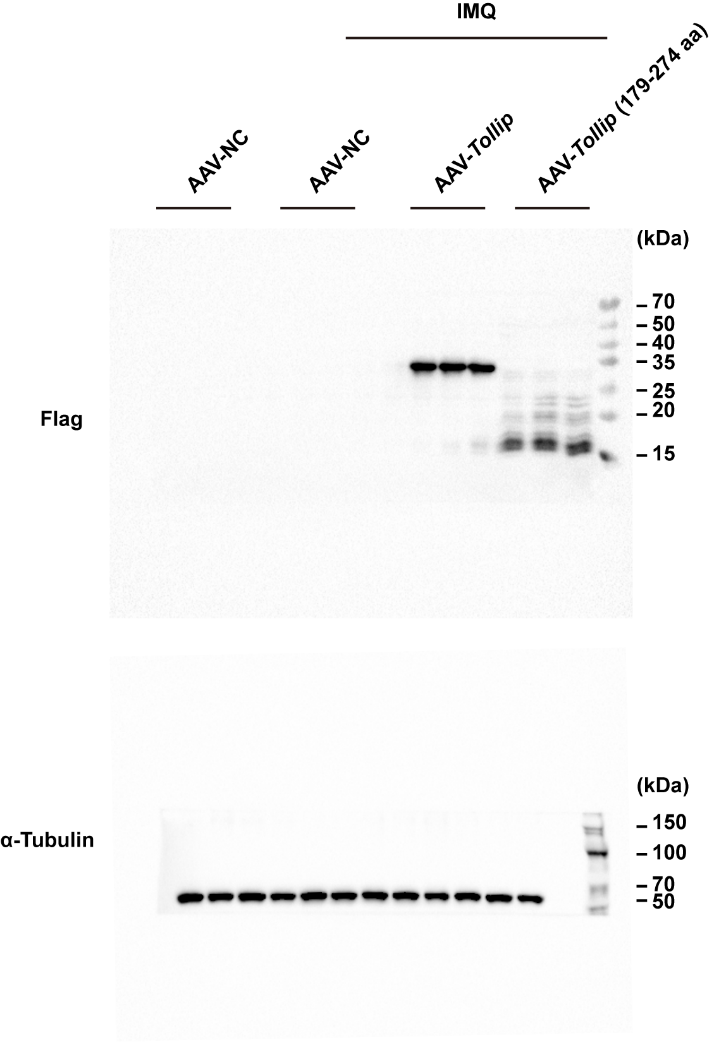


**Figure S14. Original blots for Figure S6A.**

**Table S1. Primer sequences used for human cell line qPCR experiments**

| Name | Forward primer sequence | Reverse primer sequence |
| --- | --- | --- |
| *ACTB* | 5’-CATGTACGTTGCTATCCAGGC-3’ | 5’-CTCCTTAATGTCACGCACGAT-3’ |
| *TOLLIP* | 5’-TGGGCCGACTGAACATCAC-3’ | 5’-GTGGATGACCTTATTCCAGCG-3’ |
| *KRT6* | 5’-GGGTTTCAGTGCCAACTCAG-3’ | 5’-CCAGGCCATACAGACTGCGG-3’ |
| *KRT16* | 5’-GACCGGCGGAGATGTGAAC-3’ | 5’-CTGCTCGTACTGGTCACGC-3’ |
| *KRT17* | 5’-GCCGCATCCTCAACGAGAT-3’ | 5’-CGCGGTTCAGTTCCTCTGTC-3’ |
| *IL1B* | 5’-AGCTACGAATCTCCGACCAC-3’ | 5’-CGTTATCCCATGTGTCGAAGAA-3’ |
| *IL6* | 5’-TCTGGATTCAATGAGGAGACTTG-3’ | 5’-GTTGGGTCAGGGGTGGTTAT-3’ |
| *IL23A* | 5’-CTCAGGGACAACAGTCAGTTC-3’ | 5’-ACAGGGCTATCAGGGAGCA-3’ |
| *CXCL8* | 5’-TTTTGCCAAGGAGTGCTAAAGA-3’ | 5’-AACCCTCTGCACCCAGTTTTC-3’ |
| *S100A7* | 5’-ACGTGATGACAAGATTGACAAGC-3 | 5’-GCGAGGTAATTTGTGCCCTTT-3’ |
| *S100A8* | 5’-ATGCCGTCTACAGGGATGAC-3’ | 5’-ACTGAGGACACTCGGTCTCTA-3’ |
| *S100A9* | 5’-GGTCATAGAACACATCATGGAGG-3’ | 5’-GGCCTGGCTTATGGTGGTG-3’ |
| *TNF* | 5’-GAGGCCAAGCCCTGGTATG-3’ | 5’-CGGGCCGATTGATCTCAGC-3’ |
| *CAMP* | 5’-GGCTGGTGAAGCGGTGTAT-3’ | 5’-TGGGTACAAGATTCCGCAAAAA-3’ |
| *HK2* | 5’-TGCCACCAGACTAAACTAGACG-3’ | 5’-CCCGTGCCCACAATGAGAC-3’ |
| *LDHA* | 5’-TTGACCTACGTGGCTTGGAAG-3’ | 5’-GGTAACGGAATCGGGCTGAAT-3’ |
| *PDK1* | 5’-CTGTGATACGGATCAGAAACCG-3’ | 5’-TCCACCAAACAATAAAGAGTGCT-3’ |
| *SLC2A1* | 5’-GGCCAAGAGTGTGCTAAAGAA-3’ | 5’-ACAGCGTTGATGCCAGACAG-3’ |

**Table S2. Primer sequences used for mouse qPCR experiments**

| Name | Forward primer sequence | Reverse primer sequence |
| --- | --- | --- |
| *Tuba1a* | 5’-CCTAAACAGGTTGATAGGCCAAA-3’ | 5’-CTCGCCTTCCACAGAATCCA-3’ |
| *Tollip* | 5’-CCTCAGCCCCGCTGTAATG-3’ | 5’-CAGCATCTTTGTTCCCTCTCTG-3’ |
| *Il1b* | 5’-GCAACTGTTCCTGAACTCAACT-3’ | 5’-ATCTTTTGGGGTCCGTCAACT-3’ |
| *Il6* | 5’-TAGTCCTTCCTACCCCAATTTCC-3’ | 5’-TTGGTCCTTAGCCACTCCTTC-3’ |
| *Il23a* | 5’-ATGCTGGATTGCAGAGCAGTA-3’ | 5’-ACGGGGCACATTATTTTTAGTCT-3’ |
| *S100a8* | 5’-AAATCACCATGCCCTCTACAAG-3’ | 5’-CCCACTTTTATCACCATCGCAA-3’ |
| *S100a9* | 5’-ATACTCTAGGAAGGAAGGACACC-3’ | 5’-TCCATGATGTCATTTATGAGGGC-3’ |
| *Krt16* | 5’-GGTGGCCTCTAACAGTGATCT-3’ | 5’-TGCATACAGTATCTGCCTTTGG-3’ |
| *Cxcl1* | 5’-CTGGGATTCACCTCAAGAACATC-3’ | 5’-CAGGGTCAAGGCAAGCCTC-3’ |
| *Cxcl2* | 5’-CCAACCACCAGGCTACAGG-3’ | 5’-GCGTCACACTCAAGCTCTG-3’ |
| *Pdk1* | 5’-GGACTTCGGGTCAGTGAATGC-3’ | 5’-TCCTGAGAAGATTGTCGGGGA-3’ |
| *Slc2a1* | 5’-CAGTTCGGCTATAACACTGGTG-3’ | 5’-GCCCCCGACAGAGAAGATG-3’ |
| *Camp* | 5’-GCTGTGGCGGTCACTATCAC-3’ | 5’-TGTCTAGGGACTGCTGGTTGA-3’ |
